# The macrophage in HIV-1 infection: From activation to deactivation?

**DOI:** 10.1186/1742-4690-7-33

**Published:** 2010-04-09

**Authors:** Georges Herbein, Audrey Varin

**Affiliations:** 1 Department of Virology, UPRES EA 4266 Pathogens and Inflammation, IFR 133 INSERM, Franche-Comte University, CHU Besançon, Besançon, France; 2 Cancer and Inflammation Program, Center for Cancer Research, National Cancer Institute, Frederick, MD 21702-1201, USA

## Abstract

Macrophages play a crucial role in innate and adaptative immunity in response to microorganisms and are an important cellular target during HIV-1 infection. Recently, the heterogeneity of the macrophage population has been highlighted. Classically activated or type 1 macrophages (M1) induced in particular by IFN-γ display a pro-inflammatory profile. The alternatively activated or type 2 macrophages (M2) induced by Th-2 cytokines, such as IL-4 and IL-13 express anti-inflammatory and tissue repair properties. Finally IL-10 has been described as the prototypic cytokine involved in the deactivation of macrophages (dM). Since the capacity of macrophages to support productive HIV-1 infection is known to be modulated by cytokines, this review shows how modulation of macrophage activation by cytokines impacts the capacity to support productive HIV-1 infection. Based on the activation status of macrophages we propose a model starting with M1 classically activated macrophages with accelerated formation of viral reservoirs in a context of Th1 and proinflammatory cytokines. Then IL-4/IL-13 alternatively activated M2 macrophages will enter into the game that will stop the expansion of the HIV-1 reservoir. Finally IL-10 deactivation of macrophages will lead to immune failure observed at the very late stages of the HIV-1 disease.

## Introduction

Macrophages (Ms) are the first line of defence of the organism against pathogens and, in response to the microenvironment, become differentially activated. The classical pathway of interferon-γ-dependent activation of macrophages (M1) by T helper 1 (Th1)-type responses is a well-established feature of cellular immunity to infection with HIV-1. In the presence of cytokines that are produced in a Th-2 type response, such as IL-4 and IL-13, macrophages become differentially activated (M2) and play an important role in HIV-1 pathogenesis. Although it is superficially similar to a Th2-type cytokine and is often co-induced with Th2 cytokines in the course of an immune response, it is not appropriate to classify IL-10 together with IL-4 and IL-13 as an alternative activator of macrophages. IL-10 acts on a distinct plasma membrane receptor to those for IL-4 and IL-13 [1], and its effects on macrophage gene expression are different, involving a more profound inhibition of a range of antigen-presenting and effector functions, leading to a deactivation stage of macrophages [2]. Following this line of reasoning, it seems appropriate to classify macrophages in IFN-γ classically activated macrophages (M1), IL-4/IL-13 alternatively activated macrophages (M2), and IL-10 deactivated macrophages (dM). In addition, T cells themselves are more heterogeneous than was thought originally [3,4], including not only Th0, Th1 and Th2 type cells, but also among other regulatory (Treg) and Th17 cells [5]. In addition, a wide variety of stimuli, both endogenous and exogenous, influence the susceptibility of macrophages to infection by HIV-1. The differentiation stage of monocytes/macrophages also modulates permissiveness to HIV-1: primary monocytes are less susceptible to the virus than differentiated macrophages [6-9]. The localization of macrophages in different tissues results in cells with distinct activation status and susceptibility to HIV-1 infection. Addressing the effects of macrophage differentiation and/or activation on HIV-1 replication provides some insight into the impact of specific microenvironments on macrophage infection *in vivo*. Modulation of HIV-1 replication induced by diverse stimuli have however been addressed using monocytic cell lines, primary monocytes or macrophages differentiated *in vitro *from primary monocytes. Keeping these data in mind, the present review will focus on the distinctive patterns of macrophage activation (classically activated M1, alternatively activated M2, and deactivated dM) in HIV-1 pathogenesis.

## Classical Activation of Macrophages and HIV-1 Infection

**Figure 1:**
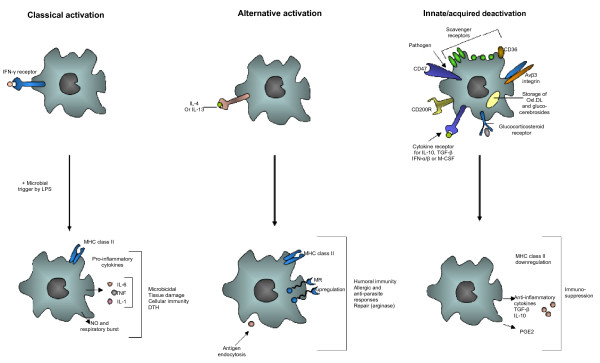
**Classical activation (M1), alternative activation (M2) and deactivation of macrophages**. Classical activation is mediated by the priming stimulus IFN-γ, followed by a microbial trigger (lipopolysaccharide, LPS). Alternative activation is mediated by IL-4 and IL-13, acting through a common receptor chain (IL-4Rα). Deactivation can be innate or acquired in origin. The uptake of apoptotic cells or lysosomal storage of host molecules generates anti-inflammatory responses. Cytokines (IL-10, TGF-β, M-CSF, IFNα/β) and glucocorticoids are potent modulators of activation. Pathogens can deactivate macrophages by various mechanisms.

Classically activated or type 1 macrophages induced in particular by IFN-γ [10], display a pro-inflammatory profile (Figure 1). In addition pro-inflammatory cytokines modulate HIV-1 replication in macrophages and could depend on the maturation and/or activation stages of monocytes/macrophages [7,8]. High levels of proinflammatory cytokines, such as tumor necrosis factor α (TNFα), interleukin (IL)-1β and IL-6 in both plasma and lymph nodes are observed from the early stages of HIV-1 infection [11-15]. The secretion of chemokines such as macrophage inflammatory protein (MIP)-1α, MIP-1β and RANTES (CCL3, CCL4 and CCL5 respectively) is increased in these patients [16,17]. Immune activation also reflects the mounting of antiviral immunity with enhanced Th1 activity and increased levels of IFNγ, IL-12, IL-2 and IL-18, especially in lymph nodes of HIV-infected subjects [18]. In addition these cytokines and their receptors have validated the importance of this pathway in cellular immunity, immunodeficiency syndromes, delayed hypersensitivity responses and tissue damage [2]. In classically activated macrophages, the following steps of the HIV-1 life cycle are modulated (Table 1).

**Table 1:** HIV-1 viral cycle in classically activated M1, alternatively activated M2 and deactivated macrophages

Viral cycle target	M1 macrophages		M2 macrophages		Deactivated macrophages	
**Entry**	***Decreased***	* CD4 downregulation: TNFα, IL1β, IFNγ, IL-2, IL-18	***Decreased***	* CXCR4 downregulation: IL-4, IL-13	***Decreased***	* CCR5 downregulation: IFNβ
		* CCR5 downregulation: TNFα, MIP-1α, MIP-1β, MCP-2, RANTES, IFNγ, GM-CSF, IL-2, IL-16, IL-15		* CCR5 downregulation IL-13	***Increased***	* CCR5 upregulation: IL-10, M-CSF
		* fusion block: RANTES		* CD4 downregulation IL-13		

**Reverse transcription**	*No effect reported*		***Decreased***	* Block of RT: IL-13	***Decreased***	* Block of RT: IL-10, IFNα/β
						* Inhibition of RT synthesis: TGFβ

**Transcription**	***Increased***	*Transactivation of HIV-1 LTR: TNF, IL-1β, IL-6, GM-CSF, IL-18	***Decreased***^+^	* Block of HIV-1 LTR transactivation: IL-4, IL-13	***Decreased***	* Block of HIV-1 LTR activation ^++^

**Post transcription**	***Decreased***	* Inhibition of viral assembly and budding: IFNγ, IL-18 (via IFNγ release),	*No effect reported*		***Decreased***	* Inhibition of viral assembly: IL-10
						* Inhibition of viral budding: IFNα/β, IL-27 (via IFNα release)

+ inhibition in differentiated macrophages

++ depends on IL-10 concentration

### Entry

HIV-1 infects monocytes/macrophages via interaction of gp120 with CD4 and either coreceptor CXCR4 or CCR5 which determines the cellular tropism [19-31]. HIV-1 envelope glycoprotein gp120 down-regulates CD4 expression in primary human macrophages through induction of endogenous TNFα [32-37]. TNFα, IL-1β and IFN-γ down-regulate both surface and total CD4 expression in primary human macrophages at the level of transcription [36,38-41]. TNFα, IFN-β, and IFN-γ inhibit R5 and R5/X4 HIV-1 entry into primary macrophages via down-regulation of both cell surface CD4 and CCR5 and via enhanced secretion of C-C chemokines, MIP-1α, MIP-1β, and RANTES [37,38,40,42-46]. An iterative pre-treatment of primary macrophages with TNFα prior to HIV infection inhibits HIV-1 replication [43]. The inhibition of HIV-1 entry into primary macrophages by TNFα involves the 75-kDa TNFR2 [43]. Another explain could be that TNFα triggers the release of granulocyte-macrophage colony-stimulating factor (GM-CSF) that has been reported to down-regulate CCR5 and subsequently block entry of R5 HIV into macrophages [47]. Interestingly, TNFR2 stimulation triggers GM-CSF secretion that has been shown to block R5 HIV-1 entry via CCR5 downregulation [47]. The inhibition of HIV-1 entry into macrophages observed following TNFα pre-treatment could be mediated via the secretion of C-C chemokines, such as RANTES, MIP-1α and MIP-1β. TNFα induces the production of RANTES, MIP-1α, and MIP-1β which in turn down-regulate cell surface CCR5 expression on primary macrophages resulting in inhibition of R5 HIV-1 entry [48-53]. In agreement with this observation, RANTES inhibits HIV-1 envelope-mediated membrane fusion in primary macrophages [54] and the activity of RANTES promoter that contains four NF-kB binding sites is up-regulated by TNFα [55]. Nevertheless, some authors report an enhancement of HIV-1 replication by RANTES in primary macrophages [27,56]. The enhancing effect of RANTES on HIV-1 infectivity may be independent of the route of virus-cell fusion and could involve two different mechanisms: one mediated via cellular activation, and the other mediated via increased virion attachment to target cells [56]. Another explanation for this discrepancy is the activation and/or differentiation status of macrophages with a more potent inhibitory effect of RANTES on monocyte-derived macrophages cultivated *in vitro *in absence of additional cytokines such as M-CSF [57].

The monocyte chemotactic protein-2 (MCP-2), but not MCP-1, has been shown to bind to CCR1, CCR2b, and CCR5 and to inhibit CD4/CCR5-mediated HIV-1 entry/replication [58]. Pretreatment of macrophages with IL-16 also inhibits R5 and R5/X4 HIV-1 replication in primary macrophages at the level of entry, although the secretion of CC-chemokines does not seem to be involved in this phenomenon [59].

IL-2 has been reported to inhibit HIV-1 replication in macrophages by down-regulating CD4 and CCR5 expression [60]. IL-15 is a Th1 cytokine produced by mononuclear phagocytes and shares many activities with IL-2, such as T-cell proliferation and activation. In addition IL-15 is more potent than IL-2 in stimulating NK cell function, including secretion of IFN-γ and of CCR5-binding chemokines [61]. *Ex vivo*, increased levels of IL-15 were detected in histocultures established from lymph nodes of individuals who were HIV positive in comparison to their uninfected counterparts [62]. Supernatants of NK cells stimulated with IL-12 and IL-15 inhibited both macrophage-tropic HIV-1_NFN-SX _and T cell-tropic HIV-1_NL4-3 _replication *in vitro*, but not dual-tropic HIV-1_89.6 _due to the use of multiple coreceptors for entry by this latter, including CXCR4, CCR5, but also CCR3 and CCR2b [24,63]. Importantly, the C-C chemokines MIP-1α, MIP-1β, and RANTES were responsible only for a fraction of the HIV-1-suppressive activity exhibited by NK cell supernatants against macrophage-tropic HIV-1. Collectively these data indicate that NK cells from normal and HIV-1^+ ^donors produce C-C chemokines and other unidentified factors that can inhibit both macrophage- and T cell-tropic HIV-1 replication *in vitro *[63].

IL-18 is a pro-inflammatory cytokine related to the IL-1 family of cytokines that plays an important role in both innate and adaptative immune responses against viruses [64,65]. Increased levels of circulating IL-18 from HIV-1 infected patients have been reported especially in the advanced and late stages of the disease [65]. IL-18 reduces cell surface expression of the HIV-1 receptor CD4 [66]. In the advanced stages of the disease, strong activation of IL-18 production along with persistent decreased production of IFN-γ, IL-12 and IL-2 may promote a Th2 immune response, which leads to persistent viral replication [65].

CD40 ligand (CD40L) is a cell surface molecule of CD4^+ ^T cells that interacts with its receptor CD40 on antigen-presenting cells (APC) to mediate thymus-dependent humoral immunity and inflammatory reactions. The stimulation of macrophages by CD40L has been shown to trigger the release of TNFα and CC-chemokines which results in down-regulation of cell surface CD4 and CCR5 and subsequent inhibition of HIV-1 entry into macrophages [17,67-69]. An in situ hybridization study showed that macrophages in lymph nodes of HIV-1 infected individuals produce MIP-1α and MIP-1β, and to a lesser extent RANTES, suggesting that HIV-1 infection might be modulated *in vivo *by activated macrophages [70]. It is interesting to note that the CD40/CD40L interaction triggers signalling through TNF receptor-associated factor 6 (TRAF6) in antigen presenting cells. TRAF6 has also been involved in innate immune responses mediated by TLR-4, such as the response to lipopolysaccharide (LPS) [68]. Like CD40L activation, LPS stimulation also induces high secretion of C-C chemokines and TNFα and inhibits infection of macrophages and CD4^+ ^T cells with R5 HIV-1 strains. Thus, during opportunistic infections, LPS might also be produced that, either directly or indirectly via TNFα production, might block HIV-1 entry into macrophages [71,72]. In human blood monocyte tissue culture-derived macrophages (TCDM), endogenous TNFα and IL-1β induced by LPS, down-regulate surface and total CD4 expression in primary macrophages [41]. Conversely, neither LPS nor TNFα/IL-1β were able to modulate surface CD4 expression on quiescent or PHA-activated lymphocytes [41]. Thus, opportunistic infections during HIV disease can result in a sustained but controlled viral production within infected macrophages.

### Transcription

TNFα has been reported to stimulate HIV-1 replication in chronically infected promonocytic U1 cell line through NF-kB activation and subsequent transactivation of the proviral LTR [73-76]. The stimulation of HIV-1 replication in U1 cell line with TNFα is mediated through the TNFR1, and not via TNFR2 [77]. Similarly, IL-1β binding to the IL-1 receptor 1, but not to the IL-1 receptor 2, stimulates HIV-1 transcription through activation of NF-kB or by an independent mechanism [75,78]. IL-1 can act alone or in synergy with IL-6 to stimulate viral replication in chronically infected promonocytic U1 cell line [78]. In addition IL-6 alone stimulates HIV-1 replication in U1 cells and primary macrophages infected with R5 AD-87 strain, but not in T cell lines [76]. Nuclear factor IL-6 (NF-IL6) is a nuclear factor that activates gene expression in response to IL-6. A consensus binding site for NF-IL6 is present in the LTR of many HIV-1 variants and the regulation of HIV-1 LTR by NF-IL6 and NF-kB/Rel transcription factors has been reported [79-81]. IL-6 stimulates HIV replication by activating viral transcription in synergy with TNFα and also by targeting a post-transcriptional step [76]. In addition, endothelial cells enhance C/EBPbeta binding activity and HIV-1 replication in macrophages. This increase in HIV-1 transcription is due in part to the production of soluble factors, such as IL-6 and also is mediated by ICAM-1 activation [82], indicating that endothelial cells, through the activation of C/EBPβ, provide a microenvironment that supports HIV-1 replication in monocytes/macrophages. The stimulation of HIV-1 replication in primary macrophages by GM-CSF is primarily due to enhanced viral transcription rather than increased viral entry [76]. GM-CSF stimulates HIV-1 replication in promonocytic U1 cells [83] and in primary human macrophages infected with the R5 HIV-1 JR-FL strain [84] by targeting HIV LTR at a site different from NF-κB [76].

*In vitro*, both acute HIV infection and incubation of the THP-1 monocytoid cell line with the accessory viral protein Nef induced expression of IL-18 [85]. Like most proinflammatory cytokines, IL-18 induces HIV expression in chronically infected monocytic cell lines via induction of the release of endogenous TNFα and IL-6 [86]. IL-18 stimulates HIV-1 replication in the chronically infected U1 monocytic cells, mediated in part via TNFα and IL-6 since the addition of anti-TNFα and anti-IL-6 antibodies reduced IL-18 increased HIV-1 production by 48% and 63%, respectively [86]. IL-18 stimulation of HIV-1 replication in U1 cells involves NF-kB and p38 MAPK activation [86].

### Posttranscription

The effect of IFN-γ on HIV-1 replication might be more complex. Pretreatment of human primary macrophages with IFN-γ before viral input has been reported either to stimulate or to inhibit HIV-1 replication [45,46,84]. In addition, IL-18 has been reported as an IFN-γ-inducing factor which inhibits HIV-1 production in PBMC through IFN-γ [66].

**Figure 2:**
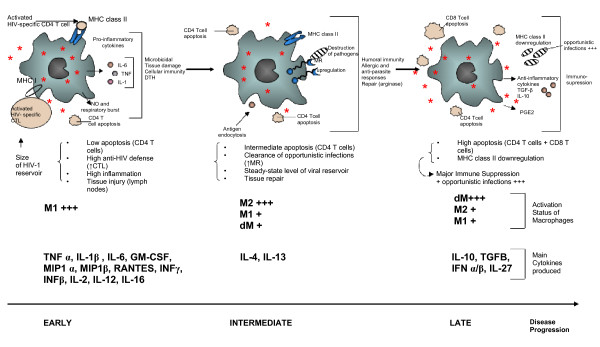
**A model of HIV-1 pathogenesis based on the activation status of macrophages**.

Altogether classically activated macrophages M1 are in contact with Th1 cytokines (IFN-γ, IL-2, IL-12), proinflammatory cytokines (TNFα, IL-1β, IL-6, IL-18) and chemokines (MIP-1α, MIP-1β, RANTES) that favor the formation of viral reservoirs with inhibition of HIV-1 entry, assembling and budding parallel to increased viral transcription within the infected macrophages (Figure 2).

## Alternative Activation of Macrophages and HIV-1 Infection

The alternatively activated or type 2 macrophages (M2) induced by Th-2 cytokines, express anti-inflammatory and tissue repair properties [2] (Figure 1). Alternative activation of macrophages is induced by IL-4 and IL-13, cytokines that are produced in a Th-2 type response, particularly during allergic, cellular and humoral responses to parasitic and selected pathogen infections. The alternative activation of macrophages is mediated by IL-4 and IL-13, acting through a common receptor chain (IL-4Rα) [87]. IL-4 is a pleiotropic cytokine produced by a subpopulation of CD4^+ ^T cells, designated Th-2 cells, and by basophiles and mast cells. IL-4 modulates other lymphoid cell activities such as regulation of the differentiation of antigen-stimulated T lymphocytes [88,89] and control of immunoglobulin class switching in B lymphocytes [90-93]. IL-13 is a cytokine secreted by activated T cells which has been shown to be a potent *in vitro *modulator of human monocytes and B cell functions [94-96]. Among its pleiotropic activities, IL-13 induces significant changes in the phenotype of human monocytes, up-regulating their expression of multiple cell surface molecules and increasing their antigen presenting capabilities. IL-4 and IL-13 upregulate expression of the mannose receptor and MHC class II molecules by macrophages which stimulate endocytosis and antigen presentation, and they induce the expression of macrophage-derived chemokine (MDC, also known as CCL22). IL-4 and IL-13 augment expression of IL-1 decoy receptor and the IL-1 receptor α-chain *in vitro *and *in vivo*, thereby counteracting the proinflammatory actions of IL-1 [97,98]. In alternatively activated macrophages, the following steps of the HIV-1 life cycle are modulated (Table 1).

### Entry

Infection of macrophages by primary R5X4 and X4 isolates of HIV-1 is inhibited by IL-4 and IL-13, an effect that is associated with down-regulation of surface CXCR4, CCR5 and CD4 expression [38,99].

### Reverse transcription

Upon cell infection by HIV-1, the reverse transcriptase copies the genomic RNA to generate the proviral DNA flanked by two LTRs [100]. IL-13 has been shown to inhibit HIV-1 replication in blood-derived monocytes and mature lung macrophages, but not in T cells [95,101]. The mechanism by which IL-13 inhibits HIV-1 is not yet clear. IL-13 has been reported either not to modulate reverse transcription [102] or to block the completion of reverse transcription in macrophages [103].

### Transcription

IL-13 has been reported to block HIV-1 replication at the level of transcription in human alveolar macrophages [102]. In fact, the state of maturation of monocytes into macrophages determines the effects of IL-4 and IL-13 on HIV-1 replication. In freshly isolated monocytes, IL-4 up-regulates the expression of both genomic and spliced HIV mRNA [104,105]. IL-4 stimulates NF-κB translocation and binding resulting in enhanced HIV RNA expression [105]. IL-4 up-regulates the expression of HIV mRNA within the first two days after infection of promonocytic U937 cells and 3 to 4 days after infection of plastic-adherent blood-derived macrophages with HIV-1 [104,106]. Conversely, IL-13 and IL-4 inhibit HIV-1 replication at the transcriptional level in differentiated macrophages, but not in peripheral blood lymphocytes [95,104,105]. In addition, exposure to IL-13 inhibits the transcription of many other cytokines in monocytes, including IL-1α, IL-1β, IL-6, TNF, and GM-CSF [96], all of which have been implicated in enhancing HIV-1 replication *in vitro *[107-110].

Altogether alternatively activated macrophages are in contact with IL-4/IL-13 producing Th2 cells that will curtail the formation of HIV-1 reservoirs in the macrophages (Figure 2).

## Deactivation of Macrophage and HIV-1 Infection

The prototypic cytokine involved in the deactivation of macrophages is IL-10. Although it is superficially similar to a Th2-type cytokine and is often co-induced with Th2 cytokines in the course of an immune response, it is not appropriate to classify IL-10 together with IL-4 and IL-13 as an alternative activator of macrophages [2]. IL-10 acts on a distinct plasma membrane receptor to those for IL-4 and IL-13 [1]. Similar to IL-10, other cytokines such as TGF-β, M-CSF and IFNα/β result in macrophage deactivation [2] with strong anti-inflammatory properties, down-regulation of MHC class II molecules on the plasma membrane (Figure 1). Deactivation of macrophages leads to immune suppression through at least two independent mechanisms: diminished MHC class II expression and increased uptake of apoptotic cells generating an anti-inflammatory response [111-115]. In deactivated macrophages, the following steps of the HIV-1 life cycle are modulated (Table 1).

### Entry

IL-10 up-regulates cell surface CCR5 expression on monocytes and thereby enhances viral entry [116]. M-CSF has been shown to favor HIV-1 replication in human macrophages, probably via an increased maturation stage and increased CCR5 expression, also resulting in enhanced viral entry [29,117]. By contrast, IFN-β inhibit R5 HIV-1 entry into primary macrophages via down-regulation of both cell surface CD4 and CCR5 and via enhanced secretion of C-C chemokines, MIP-1α, MIP-1β, and RANTES [37,40,42-46].

### Reverse transcription

IL-10 suppresses HIV-1 replication in primary human macrophages by inhibiting the initiation of reverse transcription; therefore, IL-10 mediates a virostatic latent stage in cells of the monocyte/macrophage lineage [118-120]. TGF-β inhibits the synthesis of different viral proteins especially reverse transcriptase in U1 promonocytic cells activated by phorbol ester or IL-6 [121]. Members of the APOBEC (acronym for apolipoprotein B editing catalytic polypeptide) family of cellular cytidine deaminases represent a recently identified group of proteins that provide immunity to infection by retroviruses [122-125]. The cytidine deaminases APOBEC exert anti-HIV-1 activity that is countered by the HIV-1 vif protein [122]. Tripartite motif (TRIM) proteins constitute a family of proteins that share a conserved tripartite architecture [126-128]. Interferons, especially type I IFNα/β bolster innate defence against HIV-1 via the up-regulation of APOBEC/TRIM proteins which blocks retroviral replication, especially reverse transcription [129-131].

### Transcription

High concentrations of IL-10 inhibit the production of proinflammatory cytokines such as TNFα, IL-1β, IL-6, and thereby IL-10 inhibits HIV-1 transcription [132]. By contrast, low concentrations of IL-10 have been reported to enhance HIV replication in macrophages induced by TNF-α and IL-6 via an increase in HIV mRNA accumulation and stimulation of phorbol ester-induced LTR-driven transcription that is independent of the NF-κB and Sp1 transcription factors [133].

### Posttranscription

Primary macrophages treated with IL-10 after HIV-1 inoculation show an accumulation of Gag protein suggestive of an inhibitory effect at the level of virus assembly [134]. IFNα and IFNβ reduce HIV-1 replication in primary macrophages although inhibition by IFNα has been reported to be more efficient [45,135]. Anti-HIV effects of IFNα/β are mediated by both inhibition of viral assembly and budding [136,137]. IL-27 inhibits HIV replication in monocyte-derived macrophages like IFN-α and IFN-β[138]. IL-27 suppresses the transcription of HIV-1 and preferentially inhibits HIV-1 replication in macrophages compared with CD4^+ ^T cells and activates multiple IFN-inducible genes (ISG) in macrophages like IFN-α, suggesting that IL-27 inhibits HIV-1 replication in macrophages via a mechanism similar to that of IFN-α [138-140]. Recently, of the hundred of IFN-inducible genes discovered to date, ISG15 and ISG20 have been reported to inhibit assembly and release of HIV-1 virions [141-144]. In addition the IFN-inducible tripartite motif protein TRIM22 inhibits the budding of HIV-1 with diffuse cytoplasmic distribution of Gag rather than accumulation at the plasma membrane [145]. The effects of TGF-β on the post-transcriptional steps of HIV-1 replication are more complex. In primary human macrophages, both inhibition and stimulation of HIV-1 replication have been reported following a posttreatment with TGF-β[121,146].

Altogether in deactivated macrophages, HIV-1 replication is strongly blocked at several steps of the viral life cycle especially reverse transcription, transcription and viral budding and assembly (Figure 2).

## Activation Status of Macrophages and HIV-1 Pathogenesis

Because of the various behaviours of macrophages reported (classically activated M1, alternatively activated M2, deactivated dM), we would like to present a new model that highlights the role of macrophage activation status in the modulation of viral persistence and T-cell apoptosis and could thereby further enhance our understanding of pathogenesis of HIV-mediated disease (Figure 2). We will first propose a model that applies to the monocytes/macrophages present in the blood and in the lymph nodes of HIV-1-infected patients. We will then discuss this HIV model in light of the different populations of macrophages present in distinct tissues and highlight the critical role of the microenvironment in tissues such as mucosal tissue and the central nervous system (CNS).

### Activation status of monocytes/macrophages in peripheral blood and in lymph nodes of HIV-1-infected subjects

Early in the disease, when the levels of proinflammatory cytokines, C-C chemokines and type I IFN are low and chronic immune activation is not yet predominant viral proteins are crucial for establishing a productive infection and for the activation of macrophages [147-149]. Viral proteins expressed early in the viral cycle, such as Nef, Tat, and virion-associated Vpr, activate the TNFR pathway to partially mimic TNFα biological effects, suggesting that these viral proteins can fuel the progression of the disease even in the absence of proinflammatory cytokines, especially in macrophages [9,148,150]. These viral proteins play a role in the formation of viral reservoirs in macrophages by activating transcription from the LTR and interfering with apoptotic machinery [6,151]. The classically activated macrophages M1 are in contact with high levels of Th1 cytokines (IFN-γ, IL-2, IL-12), proinflammatory cytokines (TNFα, IL-1β, IL-6, IL-18) and chemokines (MIP-1α, MIP-1β, RANTES) that favor the formation of viral reservoirs with strongly increased viral transcription and inhibition of HIV-1 entry to block superinfection within infected macrophages. In addition type I interferon production is impaired in primary HIV-1 infection with only limited inhibition of viral assembling and budding [147,152,153]. During this stage of the disease M1 macrophages are predominant, tissue injury especially in lymph nodes is observed and the rate of T-cell apoptosis is increasing [148].

At a later stage of the disease, a M1 toward M2 shift is observed with IL-4/IL-13 as pleiotropic modulators of macrophage activation that induce distinctive programmes of altered macrophage gene expression after the engagement of their specific cytokine receptors [154]. At this intermediate stage M2 macrophages appear and will favor tissue repair, the MHC class II-mediated antigen presentation and T-cell activation, the stimulation of bacterial endocytosis via the up-regulation of the mannose receptor on the cell surface [2,155]. Alternative activation of macrophages might help to favor the clearance of opportunistic infections during HIV-1 disease [156,157]. Intermediate levels of T-cell apoptosis are observed that does not totally block the production of proinflammatory cytokines [111,158]. The combination of IL-4/IL-13 cytokines and proinflammatory cytokines in the microenvironment present in the vicinity of infected macrophages will curtail the expansion of macrophage HIV-1 reservoirs [38,159].

At the onset of AIDS, T-cell apoptosis is dramatically increased and opportunistic infections are very frequent [148,158,160], resulting in an enhanced apoptotic cell clearance by IL-10-deactivated macrophages [161,162]. An imbalance in the TH1-type and TH2-type responses has been proposed to contribute to the immune dysregulation associated with HIV infection, and that progression to AIDS is dependent on a TH1/TH2 shift [163]. This hypothesis was based on the following facts: (1) progression to AIDS is characterized by loss of IL-2- and IFN-gamma production concomitant with increases in IL-10; and (2) many seronegative, HIV-exposed individuals generate strong TH1-type responses to HIV antigens. Recently, haplotypes of the IL-4 and IL-10 genes associated with AIDS progression have been reported [164,165]. In HIV-infected patients, the amount of IL-10, but not IL-4, increases significantly in patients with AIDS [166]. Opportunistic infections, especially present at the late stages of the disease, trigger IL-10 production [167] and IL-10 production from patients with AIDS has been reported to decrease *in vitro *HIV-1 replication and TNFα production [168]. In addition, IL-10 has been reported to suppress antiviral T-cell activity during persistent viral infection [169] and Tat-induced IL-10 mediates immune suppression during HIV-1 infection [170]. In addition, the IL-10 deactivated macrophages inhibit the production of proinflammatory cytokines such as TNFα and C-C chemokines that were produced abundantly due to chronic immune stimulation during the previous stages of the disease [171,172]. IL-10 inhibits HIV-1 LTR-driven gene expression in human macrophages through the induction of cyclin T1 proteolysis [173]. At the late stages of the disease the decreased levels of proinflammatory cytokines result in a strong reduction of viral transcription. In addition high expression of IFNα/β inducible proteins such as APOPEC and TRIM proteins inhibit strongly the HIV-1 reverse transcription and assembly/budding (Table 1). The deactivation of macrophages also results in a profound immune suppression resulting from the decreased expression of MHC class II expression on the plasma membrane of macrophages with diminished Ag-mediated T cell response and the depletion of both CD4+ and CD8+ T cell by accelerated apoptosis. Thus, IL-10 and type I IFN restrict strongly HIV-1 replication in macrophages parallel to the immune failure observed at the very late stages of the HIV-1 disease.

### Activation status of macrophages in mucosal tissues and in the CNS

The localization of macrophages in distinct tissues has been reported to modulate their susceptibility to HIV-1 infection. In human and macaque gastrointestinal mucosa, most attention has been focused on the small intestine, where lamina propria CD4+ T cells are prominent HIV-1 and SIV target cells and undergo profound depletion shortly after infection [174-182]. In contrast, macrophages in the gastrointestinal mucosa, unlike monocyte-derived macrophages, are rather resistant to infection with HIV-1 [183-185]. In contrast to monocytes and monocyte-macrophages, intestinal macrophages do not express many innate response receptors [186,187], are downregulated for triggering receptor expressed on monocytes (i.e., TREM-1) [188,189] and costimulatory molecules [187,190], and display markedly reduced CD4 and CCR5 cell surface protein and mRNA [191]. Thus, the striking and well-defined phenotypic and functional differences between blood monocytes and mucosal macrophages, in particular macrophages in the gastrointestinal mucosa [186,187,192], preclude the simple extrapolation from findings in HIV-1-infected monocytes to HIV-1 infection of mucosal macrophages. Human vaginal macrophages have been reported recently to support R5 virus entry in explanted vaginal mucosa, and purified vaginal macrophages support substantial levels of R5 HIV-1 replication [193]. Vaginal macrophages display the innate response receptors CD14, CD89, CD16, CD32 and CD64, and the CD4 receptor and CCR5 and CXCR4 coreceptors [193]. The difference in phenotype and HIV-1 permissiveness between vaginal and intestinal macrophages may reflect differences in the local microenvironment, since mucosa-derived cytokines, including TGF-β, regulate the phenotype and function of blood monocytes after their recruitment to the mucosa, at least in the intestinal mucosa [187]. In agreement with this hypothesis, intestinal macrophages are threefold less frequently CD4+ CCR5+ than vaginal macrophages, and yet virus is detected in intestinal macrophages, indicating low-level receptor mediated entry, but intestinal macrophages do not support viral replication suggesting a post-entry block such as described for TGF-β [193].

Macrophages of the central nervous system (CNS) are permissive to HIV-1 infection. Two models have been proposed: the Trojan horse model and the late invasive model [194]. In the Trojan horse model, the virus enters the CNS early, and replicates at low levels as a reservoir separated from the periphery. A viral phenotype that is more virulent in the context of the CNS emerges, leading to the development of disease. In the late invasion model, uncontrolled virus replication and resulting immune deficiency lead to alterations in the myeloid differentiation pathway, promoting the expansion of an activated monocyte subset that is capable of tissue invasion. The hallmark of the brain histopathology is productive infection in macrophages (perivascular macrophages and microglia) [195]. HIV encephalitis (HIVE) is characterized by monocyte/macrophage infiltration into the brain, multinucleated giant cell formation (fusion of several macrophages), and presence of microglial nodules [196]. There is little evidence for infection in neurons, endothelial cells, or macroglia (astrocytes and oligodendrocytes) [197-199]. In the Trojan horse model, it has been hypothesized that the virus enters the CNS mainly through infected monocytes and macrophages destined to become brain-resident macrophages or perivascular macrophages [200]. It is assumed that HIV-1 enters early after primary infection (at a peak of primary viremia), and HIV-1 infection persists at low levels due to the immune-privileged status of the CNS. In addition there is an uniqueness of the brain microenvironment with several anatomic/structural, physiological, and immunoregulatory mechanisms that ensure the immune priviledge of the brain, preventing recognition of foreign antigens, to minimize/deviate and block inflammatory responses [201]. Soluble anti-inflammatory molecules have been shown to play a role in immune privilege in the CNS. TGF-β has the ability to inhibit activation of macrophages, T lymphocytes, and NK cells [202], and TGF-β has been shown to possess neuroprotective capabilities [203]. Upregulation of TGF-β is observed during HIV-1 infection and is correlated with the magnitude of inflammatory responses during HIV-1 brain infection [204]. High concentrations of gangliosides downregulate expression of MHC class II on astrocytes [205] and could contribute to generally low levels of MHC class II on microglia. In contrast, a significant increase in MHC class II has been reported in the context of HIVE on activated microglia [206,207] and it is considered the best neuropathologic correlate of cognitive impairment [208]. TGF-β, IL-10, and TRAIL have been reported to contribute significantly to the CNS-DC-mediated inhibition of allo-T-cell proliferation [209] and to participate in the control of viral CNS infections [210]. In agreement with this observation, only few DC-like cells were found in perivascular spaces in SIV-infected macaques [211]. Although invasion of the CNS by HIV-1 occurs at the time of primary infection and induces a transitory inflammatory process with increased number of microglial cells, upregulation of MHC class II antigens, and local production of cytokines [212], viral replication remains very low during the asymptomatic stage of HIV-1 infection. Specific immune responses including Th2 cytokines and CTLs continuously inhibit viral replication at this stage of infection [213-216]. While HIV-1 enters the brain early following viral infection [200], detectable productive viral replication and brain macrophage infiltration occur years later and only in some infected patients [217]. The replication of HIV-1 in microglia depends on the microenvironment in the CNS. Recently, it has been reported astrocyte-mediated regulation of microglial function and its influence on the onset and the progression of neuroAIDS [218]. HIV-1, recombinant gp120, and viral transactivator Tat activate astrocytes to secrete pro-inflammatory cytokines TNFα, IL-6, and IL-1β and the pro-inflammatory chemokines MCP-1 and IP-10 [195,219-224], all of which could contribute to the overall inflammatory environment in the brain. To further contribute to the inflammatory environment in the CNS, microglia and macrophages release proinflammatory cytokines such as IL-1β and TNFα which play a role in CNS injury [225,226]. In agreement with these data, *in vivo *expression of proinflammatory cytokines in HIV-1 encephalitis has been reported and the macrophage/microglia lineage is the main cell type reported to release cytokines in HIVE [227]. Altogether, after an early and transitory stage of macrophage/microglia activation at the time of primary infection, a stage of deactivation of macrophage/microglia is observed parallel to the presence of "deactivating" cytokines such as TGF-β and IL-10 in the CNS microenvironment. In some patients, detectable productive viral infection and brain macrophage infiltration occur years later parallel to increased levels of pro-inflammatory cytokines in the context of HIVE.

### A M1/M2/Md macrophage polarization model and vice versa

Altogether, in the lymph nodes of HIV-1-infected patients a shift from activated to deactivated macrophages throughout the disease is observed parallel to a Th1 pro-inflammatory/Th2 anti-inflammatory switch. In some tissue such as the intestinal mucosal tissue, the macrophages are mostly in a deactivated stage with a local microenvironment curtailing the viral replication through the release of anti-inflammatory cytokines such as TGF-β. In contrast to the intestinal mucosa, macrophages from the vaginal mucosa are more permissive to HIV-1 replication and are activated by proinflammatory cytokines. In the CNS of HIV-infected patients, the macrophage/microglia are mostly deactivated under the control of cytokines such as TGF-β, although in some cases HIVE occurs parallel to the production of proinflammatory cytokines and high viral production at advanced stage of the disease. Thus the shift of macrophage/microglia from activation to deactivation and vice-versa depends on the tissue infected by HIV-1 and on the local microenvironment. In agreement with this hypothesis, the reversion of M2/Md macrophages to M1 polarization has been recently reported *in vitro*, and was associated with a renewed capacity to support HIV-1 replication [228]. M1/M2/Md macrophage polarization may represent a mechanism that allows macrophages to cycle between productive and latent HIV-1 infection and vice-versa, parallel to the critical role of the tissue microenvironment which can drive the macrophage polarization either way and thereby can modulate HIV-1 replication specifically in distinct tissues at different stages of the disease.

## Conclusion

The concept of macrophage heterogeneity and differentiation has been recently highlighted by the description of at least three types of macrophage activation: M1, M2 and deactivated macrophages. Based on the activation status of macrophages we propose a model starting with M1 classically activated macrophages with accelerated formation of viral reservoirs in a context of Th1 and proinflammatory cytokines. Then IL-4/IL-13 alternatively activated M2 macrophages will enter into the game that will be concomitant to tissue repair, enhanced MHC class II-mediated antigen presentation, increased T-cell activation, and enhanced clearance of opportunistic pathogens via bacterial endocytosis. At this stage of the disease, the expansion of the HIV-1 reservoir in IL-4/IL-13 alternatively activated M2 macrophages will be stopped [228]. The M2 macrophages will be in the vicinity of Th2 cells with the appearance of IL-10 deactivation of macrophages leading to immune failure observed at the very late stages of the HIV-1 disease with diminished Ag-mediated T cell response and accelerated depletion of both CD4+ and CD8+ T cells by apoptosis [229]. A better understanding of the macrophage activation status during the progression of HIV-1 infection could lead to the development of new therapeutic approaches.

## Competing interests

The authors declare that they have no competing interests.

## Authors' contributions

GH was responsible for drafting and revising the manuscript as well as organizing the content. AV assisted in revising the manuscript.

## References

[B1] Moore KW de Waal Malefyt R Coffman RL O'Garra A Interleukin-10 and the interleukin-10 receptor Annu Rev Immunol 2001 19 683 765 10.1146/annurev.immunol.19.1.683 11244051

[B2] Gordon S Alternative activation of macrophages Nat Rev Immunol 2003 3 23 35 10.1038/nri978 12511873

[B3] O'Garra A Arai N The molecular basis of T helper 1 and T helper 2 cell differentiation Trends Cell Biol 2000 10 542 550 10.1016/S0962-8924(00)01856-0 11121747

[B4] Colonna M Can we apply the TH1-TH2 paradigm to all lymphocytes? Nat Immunol 2001 2 899 900 10.1038/ni1001-899 11577341

[B5] Locksley RM Nine lives: plasticity among T helper cell subsets J Exp Med 2009 206 1643 1646 10.1084/jem.20091442 19635860 PMC2722180

[B6] Coleman CM Wu L HIV interactions with monocytes and dendritic cells: viral latency and reservoirs Retrovirology 2009 6 51 10.1186/1742-4690-6-51 19486514 PMC2697150

[B7] Rich EA Chen IS Zack JA Leonard ML O'Brien WA Increased susceptibility of differentiated mononuclear phagocytes to productive infection with human immunodeficiency virus-1 (HIV-1) J Clin Invest 1992 89 176 183 10.1172/JCI115559 1370293 PMC442834

[B8] Sonza S Maerz A Deacon N Meanger J Mills J Crowe S Human immunodeficiency virus type 1 replication is blocked prior to reverse transcription and integration in freshly isolated peripheral blood monocytes J Virol 1996 70 3863 3869 8648722 10.1128/jvi.70.6.3863-3869.1996 PMC190263

[B9] Yu W Wang Y Shaw CA Qin XF Rice AP Induction of the HIV-1 Tat co-factor cyclin T1 during monocyte differentiation is required for the regulated expression of a large portion of cellular mRNAs Retrovirology 2006 3 32 10.1186/1742-4690-3-32 16764723 PMC1557533

[B10] Dalton DK Pitts-Meek S Keshav S Figari IS Bradley A Stewart TA Multiple defects of immune cell function in mice with disrupted interferon-gamma genes Science 1993 259 1739 1742 10.1126/science.8456300 8456300

[B11] Weiss L Haeffner-Cavaillon N Laude M Gilquin J Kazatchkine MD HIV infection is associated with the spontaneous production of interleukin-1 (IL-1) in vivo and with an abnormal release of IL-1 alpha in vitro AIDS 1989 3 695 699 10.1097/00002030-198911000-00002 2515876

[B12] Molina JM Scadden DT Byrn R Dinarello CA Groopman JE Production of tumor necrosis factor alpha and interleukin 1 beta by monocytic cells infected with human immunodeficiency virus J Clin Invest 1989 84 733 737 10.1172/JCI114230 2474573 PMC329713

[B13] Emilie D Peuchmaur M Maillot MC Crevon MC Brousse N Delfraissy JF Dormont J Galanaud P Production of interleukins in human immunodeficiency virus-1-replicating lymph nodes J Clin Invest 1990 86 148 159 10.1172/JCI114678 2114424 PMC296702

[B14] Birx DL Redfield RR Tencer K Fowler A Burke DS Tosato G Induction of interleukin-6 during human immunodeficiency virus infection Blood 1990 76 2303 2310 2257304

[B15] Lafeuillade A Poizot-Martin I Quilichini R Gastaut JA Kaplanski S Farnarier C Mege JL Bongrand P Increased interleukin-6 production is associated with disease progression in HIV infection AIDS 1991 5 1139 1140 10.1097/00002030-199109000-00014 1930778

[B16] Canque B Rosenzwajg M Gey A Tartour E Fridman WH Gluckman JC Macrophage inflammatory protein-1alpha is induced by human immunodeficiency virus infection of monocyte-derived macrophages Blood 1996 87 2011 2019 8634452

[B17] Cotter RL Zheng J Che M Niemann D Liu Y He J Thomas E Gendelman HE Regulation of human immunodeficiency virus type 1 infection, beta-chemokine production, and CCR5 expression in CD40L-stimulated macrophages: immune control of viral entry J Virol 2001 75 4308 4320 10.1128/JVI.75.9.4308-4320.2001 11287580 PMC114176

[B18] Lisziewicz J Gabrilovich DI Varga G Xu J Greenberg PD Arya SK Bosch M Behr JP Lori F Induction of potent human immunodeficiency virus type 1-specific T-cell-restricted immunity by genetically modified dendritic cells J Virol 2001 75 7621 7628 10.1128/JVI.75.16.7621-7628.2001 11462034 PMC114997

[B19] Alkhatib G Combadiere C Broder CC Feng Y Kennedy PE Murphy PM Berger EA CC CKR5: a RANTES, MIP-1alpha, MIP-1beta receptor as a fusion cofactor for macrophage-tropic HIV-1 Science 1996 272 1955 1958 10.1126/science.272.5270.1955 8658171

[B20] Choe H Farzan M Sun Y Sullivan N Rollins B Ponath PD Wu L Mackay CR LaRosa G Newman W Gerard N Gerard C Sodroski J The beta-chemokine receptors CCR3 and CCR5 facilitate infection by primary HIV-1 isolates Cell 1996 85 1135 1148 10.1016/S0092-8674(00)81313-6 8674119

[B21] Cocchi F DeVico AL Garzino-Demo A Arya SK Gallo RC Lusso P Identification of RANTES, MIP-1 alpha, and MIP-1 beta as the major HIV-suppressive factors produced by CD8+ T cells Science 1995 270 1811 1815 10.1126/science.270.5243.1811 8525373

[B22] Collin M Herbein G Montaner L Gordon S PCR analysis of HIV1 infection of macrophages: virus entry is CD4-dependent Res Virol 1993 144 13 19 10.1016/S0923-2516(06)80006-3 8446772

[B23] Deng H Liu R Ellmeier W Choe S Unutmaz D Burkhart M Di Marzio P Marmon S Sutton RE Hill CM Davis CB Peiper SC Schall TJ Littman DR Landau NR Identification of a major co-receptor for primary isolates of HIV-1 Nature 1996 381 661 666 10.1038/381661a0 8649511

[B24] Doranz BJ Rucker J Yi Y Smyth RJ Samson M Peiper SC Parmentier M Collman RG Doms RW A dual-tropic primary HIV-1 isolate that uses fusin and the beta-chemokine receptors CKR-5, CKR-3, and CKR-2b as fusion cofactors Cell 1996 85 1149 1158 10.1016/S0092-8674(00)81314-8 8674120

[B25] Feng Y Broder CC Kennedy PE Berger EA HIV-1 entry cofactor: functional cDNA cloning of a seven-transmembrane, G protein-coupled receptor Science 1996 272 872 877 10.1126/science.272.5263.872 8629022

[B26] Liu R Paxton WA Choe S Ceradini D Martin SR Horuk R MacDonald ME Stuhlmann H Koup RA Landau NR Homozygous defect in HIV-1 coreceptor accounts for resistance of some multiply-exposed individuals to HIV-1 infection Cell 1996 86 367 377 10.1016/S0092-8674(00)80110-5 8756719

[B27] Schmidtmayerova H Sherry B Bukrinsky M Chemokines and HIV replication Nature 1996 382 767 10.1038/382767a0 8752270

[B28] Verani A Scarlatti G Comar M Tresoldi E Polo S Giacca M Lusso P Siccardi AG Vercelli D C-C chemokines released by lipopolysaccharide (LPS)-stimulated human macrophages suppress HIV-1 infection in both macrophages and T cells J Exp Med 1997 185 805 816 10.1084/jem.185.5.805 9120386 PMC2196157

[B29] Wang J Roderiquez G Oravecz T Norcross MA Cytokine regulation of human immunodeficiency virus type 1 entry and replication in human monocytes/macrophages through modulation of CCR5 expression J Virol 1998 72 7642 7647 9696868 10.1128/jvi.72.9.7642-7647.1998 PMC110028

[B30] Rossi F Querido B Nimmagadda M Cocklin S Navas-Martin S Martin-Garcia J The V1-V3 region of a brain-derived HIV-1 envelope glycoprotein determines macrophage tropism, low CD4 dependence, increased fusogenicity and altered sensitivity to entry inhibitors Retrovirology 2008 5 89 10.1186/1742-4690-5-89 18837996 PMC2576352

[B31] Dunfee RL Thomas ER Gabuzda D Enhanced macrophage tropism of HIV in brain and lymphoid tissues is associated with sensitivity to the broadly neutralizing CD4 binding site antibody b12 Retrovirology 2009 6 69 10.1186/1742-4690-6-69 19619305 PMC2717910

[B32] Bergamini A Faggioli E Bolacchi F Gessani S Cappannoli L Uccella I Demin F Capozzi M Cicconi R Placido R Vendetti S Colizzi GM Rocchi G Enhanced production of tumor necrosis factor-alpha and interleukin-6 due to prolonged response to lipopolysaccharide in human macrophages infected in vitro with human immunodeficiency virus type 1 J Infect Dis 1999 179 832 842 10.1086/314662 10068578

[B33] Choe W Volsky DJ Potash MJ Induction of rapid and extensive beta-chemokine synthesis in macrophages by human immunodeficiency virus type 1 and gp120, independently of their coreceptor phenotype J Virol 2001 75 10738 10745 10.1128/JVI.75.22.10738-10745.2001 11602715 PMC114655

[B34] Clouse KA Cosentino LM Weih KA Pyle SW Robbins PB Hochstein HD Natarajan V Farrar WL The HIV-1 gp120 envelope protein has the intrinsic capacity to stimulate monokine secretion J Immunol 1991 147 2892 2901 1918997

[B35] Herbein G Keshav S Collin M Montaner LJ Gordon S HIV-1 induces tumour necrosis factor and IL-1 gene expression in primary human macrophages independent of productive infection Clin Exp Immunol 1994 95 442 449 7511077 10.1111/j.1365-2249.1994.tb07016.x PMC1535095

[B36] Karsten V Gordon S Kirn A Herbein G HIV-1 envelope glycoprotein gp120 down-regulates CD4 expression in primary human macrophages through induction of endogenous tumour necrosis factor-alpha Immunology 1996 88 55 60 10.1046/j.1365-2567.1996.d01-648.x 8707350 PMC1456460

[B37] Merrill JE Koyanagi Y Chen IS Interleukin-1 and tumor necrosis factor alpha can be induced from mononuclear phagocytes by human immunodeficiency virus type 1 binding to the CD4 receptor J Virol 1989 63 4404 4408 2789293 10.1128/jvi.63.10.4404-4408.1989 PMC251058

[B38] Bailer RT Lee B Montaner LJ IL-13 and TNF-alpha inhibit dual-tropic HIV-1 in primary macrophages by reduction of surface expression of CD4, chemokine receptors CCR5, CXCR4 and post-entry viral gene expression Eur J Immunol 2000 30 1340 1349 10.1002/(SICI)1521-4141(200005)30:5<1340::AID-IMMU1340>3.0.CO;2-L 10820380

[B39] Faltynek CR Finch LR Miller P Overton WR Treatment with recombinant IFN-gamma decreases cell surface CD4 levels on peripheral blood monocytes and on myelomonocyte cell lines J Immunol 1989 142 500 508 2492048

[B40] Hariharan D Douglas SD Lee B Lai JP Campbell DE Ho WZ Interferon-gamma upregulates CCR5 expression in cord and adult blood mononuclear phagocytes Blood 1999 93 1137 1144 9949155

[B41] Herbein G Doyle AG Montaner LJ Gordon S Lipopolysaccharide (LPS) down-regulates CD4 expression in primary human macrophages through induction of endogenous tumour necrosis factor (TNF) and IL-1 beta Clin Exp Immunol 1995 102 430 437 7586702 10.1111/j.1365-2249.1995.tb03801.x PMC1553416

[B42] Cremer I Vieillard V De Maeyer E Retrovirally mediated IFN-beta transduction of macrophages induces resistance to HIV, correlated with up-regulation of RANTES production and down-regulation of C-C chemokine receptor-5 expression J Immunol 2000 164 1582 1587 10640778 10.4049/jimmunol.164.3.1582

[B43] Herbein G Montaner LJ Gordon S Tumor necrosis factor alpha inhibits entry of human immunodeficiency virus type 1 into primary human macrophages: a selective role for the 75-kilodalton receptor J Virol 1996 70 7388 7397 8892857 10.1128/jvi.70.11.7388-7397.1996 PMC190806

[B44] Hewson TJ Logie JJ Simmonds P Howie SE A CCR5-dependent novel mechanism for type 1 HIV gp120 induced loss of macrophage cell surface CD4 J Immunol 2001 166 4835 4842 11290759 10.4049/jimmunol.166.8.4835

[B45] Meylan PR Guatelli JC Munis JR Richman DD Kornbluth RS Mechanisms for the inhibition of HIV replication by interferons-alpha, -beta, and -gamma in primary human macrophages Virology 1993 193 138 148 10.1006/viro.1993.1110 7679856

[B46] Zaitseva M Lee S Lapham C Taffs R King L Romantseva T Manischewitz J Golding H Interferon gamma and interleukin 6 modulate the susceptibility of macrophages to human immunodeficiency virus type 1 infection Blood 2000 96 3109 3117 11049991

[B47] Di Marzio P Tse J Landau NR Chemokine receptor regulation and HIV type 1 tropism in monocyte-macrophages AIDS Res Hum Retroviruses 1998 14 129 138 10.1089/aid.1998.14.129 9462923

[B48] Capobianchi MR Abbate I Antonelli G Turriziani O Dolei A Dianzani F Inhibition of HIV type 1 BaL replication by MIP-1alpha, MIP-1beta, and RANTES in macrophages AIDS Res Hum Retroviruses 1998 14 233 240 10.1089/aid.1998.14.233 9491913

[B49] Coffey MJ Woffendin C Phare SM Strieter RM Markovitz DM RANTES inhibits HIV-1 replication in human peripheral blood monocytes and alveolar macrophages Am J Physiol 1997 272 L1025 1029 9176270 10.1152/ajplung.1997.272.5.L1025

[B50] Jiang Y Jolly PE Effect of beta-chemokines on human immunodeficiency virus type 1 replication, binding, uncoating, and CCR5 receptor expression in human monocyte-derived macrophages J Hum Virol 1999 2 123 132 10413363

[B51] Lane BR Markovitz DM Woodford NL Rochford R Strieter RM Coffey MJ TNF-alpha inhibits HIV-1 replication in peripheral blood monocytes and alveolar macrophages by inducing the production of RANTES and decreasing C-C chemokine receptor 5 (CCR5) expression J Immunol 1999 163 3653 3661 10490959

[B52] Ylisastigui L Amzazi S Bakri Y Vizzavona J Vita C Gluckman JC Benjouad A Effect of RANTES on the infection of monocyte-derived primary macrophages by human immunodeficiency virus type 1 and type 2 Biomedicine & Pharmacotherapy 1998 52 447 453 10.1016/S0753-3322(99)80023-7 9921414

[B53] Grainger DJ Lever AM Blockade of chemokine-induced signalling inhibits CCR5-dependent HIV infection in vitro without blocking gp120/CCR5 interaction Retrovirology 2005 2 23 10.1186/1742-4690-2-23 15807900 PMC1082716

[B54] Stantchev TS Broder CC Consistent and significant inhibition of human immunodeficiency virus type 1 envelope-mediated membrane fusion by beta-chemokines (RANTES) in primary human macrophages J Infect Dis 2000 182 68 78 10.1086/315700 10882583

[B55] Moriuchi H Moriuchi M Fauci AS Nuclear factor-kappa B potently up-regulates the promoter activity of RANTES, a chemokine that blocks HIV infection J Immunol 1997 158 3483 3491 9120310

[B56] Gordon CJ Muesing MA Proudfoot AE Power CA Moore JP Trkola A Enhancement of human immunodeficiency virus type 1 infection by the CC-chemokine RANTES is independent of the mechanism of virus-cell fusion J Virol 1999 73 684 694 9847374 10.1128/jvi.73.1.684-694.1999 PMC103875

[B57] Amzazi S Ylisastigui L Bakri Y Rabehi L Gattegno L Parmentier M Gluckman JC Benjouad A The inhibitory effect of RANTES on the infection of primary macrophages by R5 human immunodeficiency virus type-1 depends on the macrophage activation state Virology 1998 252 96 105 10.1006/viro.1998.9452 9875320

[B58] Gong W Howard OM Turpin JA Grimm MC Ueda H Gray PW Raport CJ Oppenheim JJ Wang JM Monocyte chemotactic protein-2 activates CCR5 and blocks CD4/CCR5-mediated HIV-1 entry/replication J Biol Chem 1998 273 4289 4292 10.1074/jbc.273.8.4289 9468473

[B59] Truong MJ Darcissac EC Hermann E Dewulf J Capron A Bahr GM Interleukin-16 inhibits human immunodeficiency virus type 1 entry and replication in macrophages and in dendritic cells J Virol 1999 73 7008 7013 10400800 10.1128/jvi.73.8.7008-7013.1999 PMC112787

[B60] Kutza J Hayes MP Clouse KA Interleukin-2 inhibits HIV-1 replication in human macrophages by modulating expression of CD4 and CC-chemokine receptor-5 Aids 1998 12 F59 64 10.1097/00002030-199808000-00001 9631132

[B61] Rodriguez AR Arulanandam BP Hodara VL McClure HM Cobb EK Salas MT White R Murthy KK Influence of interleukin-15 on CD8+ natural killer cells in human immunodeficiency virus type 1-infected chimpanzees J Gen Virol 2007 88 641 651 10.1099/vir.0.82154-0 17251583

[B62] Biancotto A Grivel JC Iglehart SJ Vanpouille C Lisco A Sieg SF Debernardo R Garate K Rodriguez B Margolis LB Lederman MM Abnormal activation and cytokine spectra in lymph nodes of people chronically infected with HIV-1 Blood 2007 109 4272 4279 10.1182/blood-2006-11-055764 17289812 PMC1885500

[B63] Fehniger TA Herbein G Yu H Para MI Bernstein ZP O'Brien WA Caligiuri MA Natural killer cells from HIV-1+ patients produce C-C chemokines and inhibit HIV-1 infection J Immunol 1998 161 6433 6438 9834136

[B64] Ahmad R Sindhu ST Toma E Morisset R Ahmad A Elevated levels of circulating interleukin-18 in human immunodeficiency virus-infected individuals: role of peripheral blood mononuclear cells and implications for AIDS pathogenesis J Virol 2002 76 12448 12456 10.1128/JVI.76.24.12448-12456.2002 12438570 PMC136707

[B65] Torre D Pugliese A Interleukin-18: a proinflammatory cytokine in HIV-1 infection Curr HIV Res 2006 4 423 430 10.2174/157016206778559993 17073617

[B66] Choi HJ Dinarello CA Shapiro L Interleukin-18 inhibits human immunodeficiency virus type 1 production in peripheral blood mononuclear cells J Infect Dis 2001 184 560 568 10.1086/322805 11494162

[B67] di Marzio P Mariani R Lui R Thomas EK Landau NR Soluble CD40 ligand induces beta-chemokine production by macrophages and resistance to HIV-1 entry Cytokine 2000 12 1489 1495 10.1006/cyto.1999.0594 11023663

[B68] Kornbluth RS The emerging role of CD40 ligand in HIV infection J Leukoc Biol 2000 68 373 382 10985254

[B69] Kornbluth RS Kee K Richman DD CD40 ligand (CD154) stimulation of macrophages to produce HIV-1-suppressive beta-chemokines Proc Natl Acad Sci USA 1998 95 5205 5210 10.1073/pnas.95.9.5205 9560254 PMC20239

[B70] Tedla N Palladinetti P Kelly M Kumar RK DiGirolamo N Chattophadhay U Cooke B Truskett P Dwyer J Wakefield D Lloyd A Chemokines and T lymphocyte recruitment to lymph nodes in HIV infection Am J Pathol 1996 148 1367 1373 8623908 PMC1861577

[B71] Alfano M Schmidtmayerova H Amella CA Pushkarsky T Bukrinsky M The B-oligomer of pertussis toxin deactivates CC chemokine receptor 5 and blocks entry of M-tropic HIV-1 strains J Exp Med 1999 190 597 605 10.1084/jem.190.5.597 10477545 PMC2195621

[B72] Zybarth G Reiling N Schmidtmayerova H Sherry B Bukrinsky M Activation-induced resistance of human macrophages to HIV-1 infection in vitro J Immunol 1999 162 400 406 9886413

[B73] Palmieri C Trimboli F Puca A Fiume G Scala G Quinto I Inhibition of HIV-1 replication in primary human monocytes by the IkappaB-alphaS32/36A repressor of NF-kappaB Retrovirology 2004 1 45 10.1186/1742-4690-1-45 15613239 PMC544834

[B74] Duh EJ Maury WJ Folks TM Fauci AS Rabson AB Tumor necrosis factor alpha activates human immunodeficiency virus type 1 through induction of nuclear factor binding to the NF-kappa B sites in the long terminal repeat Proc Natl Acad Sci USA 1989 86 5974 5978 10.1073/pnas.86.15.5974 2762307 PMC297754

[B75] Osborn L Kunkel S Nabel GJ Tumor necrosis factor alpha and interleukin 1 stimulate the human immunodeficiency virus enhancer by activation of the nuclear factor kappa B Proc Natl Acad Sci USA 1989 86 2336 2340 10.1073/pnas.86.7.2336 2494664 PMC286907

[B76] Poli G Bressler P Kinter A Duh E Timmer WC Rabson A Justement JS Stanley S Fauci AS Interleukin 6 induces human immunodeficiency virus expression in infected monocytic cells alone and in synergy with tumor necrosis factor alpha by transcriptional and post-transcriptional mechanisms J Exp Med 1990 172 151 158 10.1084/jem.172.1.151 2193094 PMC2188185

[B77] Butera ST Roberts BD Leung K Nabel GJ Folks TM Tumor necrosis factor receptor expression and signal transduction in HIV-1-infected cells Aids 1993 7 911 918 10.1097/00002030-199307000-00002 8395188

[B78] Poli G Kinter AL Fauci AS Interleukin 1 induces expression of the human immunodeficiency virus alone and in synergy with interleukin 6 in chronically infected U1 cells: inhibition of inductive effects by the interleukin 1 receptor antagonist Proc Natl Acad Sci USA 1994 91 108 112 10.1073/pnas.91.1.108 7506410 PMC42895

[B79] Ruocco MR Chen X Ambrosino C Dragonetti E Liu W Mallardo M De Falco G Palmieri C Franzoso G Quinto I Venuta S Scala G Regulation of HIV-1 long terminal repeats by interaction of C/EBP(NF-IL6) and NF-kappaB/Rel transcription factors J Biol Chem 1996 271 22479 22486 10.1074/jbc.271.37.22479 8798413

[B80] Tesmer VM Rajadhyaksha A Babin J Bina M NF-IL6-mediated transcriptional activation of the long terminal repeat of the human immunodeficiency virus type 1 Proc Natl Acad Sci USA 1993 90 7298 7302 10.1073/pnas.90.15.7298 8346247 PMC47124

[B81] Yang Y Tesmer VM Bina M Regulation of HIV-1 transcription in activated monocyte macrophages Virology 2002 299 256 265 10.1006/viro.2001.1530 12202228

[B82] Lee ES Zhou H Henderson AJ Endothelial cells enhance human immunodeficiency virus type 1 replication in macrophages through a C/EBP-dependent mechanism J Virol 2001 75 9703 9712 10.1128/JVI.75.20.9703-9712.2001 11559803 PMC114542

[B83] Folks TM Justement J Kinter A Dinarello CA Fauci AS Cytokine-induced expression of HIV-1 in a chronically infected promonocyte cell line Science 1987 238 800 802 10.1126/science.3313729 3313729

[B84] Koyanagi Y O'Brien WA Zhao JQ Golde DW Gasson JC Chen IS Cytokines alter production of HIV-1 from primary mononuclear phagocytes Science 1988 241 1673 1675 10.1126/science.3047875 3047875

[B85] Pugliese A Vidotto V Beltramo T Torre D Regulation of interleukin-18 by THP-1 monocytoid cells stimulated with HIV-1 and Nef viral protein Eur Cytokine Netw 2005 16 186 190 16266857

[B86] Shapiro L Puren AJ Barton HA Novick D Peskind RL Shenkar R Gu Y Su MS Dinarello CA Interleukin 18 stimulates HIV type 1 in monocytic cells Proc Natl Acad Sci USA 1998 95 12550 12555 10.1073/pnas.95.21.12550 9770523 PMC22868

[B87] Zurawski SM Vega F Jr Huyghe B Zurawski G Receptors for interleukin-13 and interleukin-4 are complex and share a novel component that functions in signal transduction EMBO J 1993 12 2663 2670 8101483 10.1002/j.1460-2075.1993.tb05927.x PMC413514

[B88] Hsieh CS Heimberger AB Gold JS O'Garra A Murphy KM Differential regulation of T helper phenotype development by interleukins 4 and 10 in an alpha beta T-cell-receptor transgenic system Proc Natl Acad Sci USA 1992 89 6065 6069 10.1073/pnas.89.13.6065 1385868 PMC49438

[B89] Seder RA Paul WE Davis MM Fazekas de St Groth B The presence of interleukin 4 during in vitro priming determines the lymphokine-producing potential of CD4+ T cells from T cell receptor transgenic mice J Exp Med 1992 176 1091 1098 10.1084/jem.176.4.1091 1328464 PMC2119379

[B90] Coffman RL Ohara J Bond MW Carty J Zlotnik A Paul WE B cell stimulatory factor-1 enhances the IgE response of lipopolysaccharide-activated B cells J Immunol 1986 136 4538 4541 3486902

[B91] Gascan H Gauchat JF Roncarolo MG Yssel H Spits H de Vries JE Human B cell clones can be induced to proliferate and to switch to IgE and IgG4 synthesis by interleukin 4 and a signal provided by activated CD4+ T cell clones J Exp Med 1991 173 747 750 10.1084/jem.173.3.747 1997653 PMC2118815

[B92] Vitetta ES Ohara J Myers CD Layton JE Krammer PH Paul WE Serological, biochemical, and functional identity of B cell-stimulatory factor 1 and B cell differentiation factor for IgG1 J Exp Med 1985 162 1726 1731 10.1084/jem.162.5.1726 3932582 PMC2187936

[B93] Varin A Gordon S Alternative activation of macrophages: Immune function and cellular biology Immunobiology 2009 214 630 41 10.1016/j.imbio.2008.11.009 19264378

[B94] de Waal Malefyt R Figdor CG Huijbens R Mohan-Peterson S Bennett B Culpepper J Dang W Zurawski G de Vries JE Effects of IL-13 on phenotype, cytokine production, and cytotoxic function of human monocytes. Comparison with IL-4 and modulation by IFN-gamma or IL-10 J Immunol 1993 151 6370 6381 7902377

[B95] Montaner LJ Doyle AG Collin M Herbein G Illei P James W Minty A Caput D Ferrara P Gordon S Interleukin 13 inhibits human immunodeficiency virus type 1 production in primary blood-derived human macrophages in vitro J Exp Med 1993 178 743 747 10.1084/jem.178.2.743 8101865 PMC2191127

[B96] Zurawski G de Vries JE Interleukin 13, an interleukin 4-like cytokine that acts on monocytes and B cells, but not on T cells Immunol Today 1994 15 19 26 10.1016/0167-5699(94)90021-3 7907877

[B97] Mantovani A Locati M Vecchi A Sozzani S Allavena P Decoy receptors: a strategy to regulate inflammatory cytokines and chemokines Trends Immunol 2001 22 328 336 10.1016/S1471-4906(01)01941-X 11377293

[B98] Fenton MJ Buras JA Donnelly RP IL-4 reciprocally regulates IL-1 and IL-1 receptor antagonist expression in human monocytes J Immunol 1992 149 1283 1288 1386862

[B99] Wang J Guan E Roderiquez G Calvert V Alvarez R Norcross MA Role of tyrosine phosphorylation in ligand-independent sequestration of CXCR4 in human primary monocytes-macrophages J Biol Chem 2001 276 49236 49243 10.1074/jbc.M108523200 11668182

[B100] Mougel M Houzet L Darlix JL When is it time for reverse transcription to start and go? Retrovirology 2009 6 24 10.1186/1742-4690-6-24 19261185 PMC2656454

[B101] Denis M Ghadirian E Interleukin 13 and interleukin 4 protect bronchoalveolar macrophages from productive infection with human immunodeficiency virus type 1 AIDS Res Hum Retroviruses 1994 10 795 802 10.1089/aid.1994.10.1619 7986585

[B102] Hatch WC Freedman AR Boldt-Houle DM Groopman JE Terwilliger EF Differential effects of interleukin-13 on cytomegalovirus and human immunodeficiency virus infection in human alveolar macrophages Blood 1997 89 3443 3450 9129052

[B103] Montaner LJ Bailer RT Gordon S IL-13 acts on macrophages to block the completion of reverse transcription, inhibit virus production, and reduce virus infectivity J Leukoc Biol 1997 62 126 132 9226003 10.1002/jlb.62.1.126

[B104] Mikovits JA Meyers AM Ortaldo JR Minty A Caput D Ferrara P Ruscetti FW IL-4 and IL-13 have overlapping but distinct effects on HIV production in monocytes J Leukoc Biol 1994 56 340 346 7916030 10.1002/jlb.56.3.340

[B105] Naif HM Li S Ho-Shon M Mathijs JM Williamson P Cunningham AL The state of maturation of monocytes into macrophages determines the effects of IL-4 and IL-13 on HIV replication J Immunol 1997 158 501 511 8977228

[B106] Naif H Ho-Shon M Chang J Cunningham AL Molecular mechanisms of IL-4 effect on HIV expression in promonocytic cell lines and primary human monocytes J Leukoc Biol 1994 56 335 339 8083605 10.1002/jlb.56.3.335

[B107] Kobayashi N Hamamoto Y Koyanagi Y Chen IS Yamamoto N Effect of interleukin-1 on the augmentation of human immunodeficiency virus gene expression Biochem Biophys Res Commun 1989 165 715 721 10.1016/S0006-291X(89)80025-7 2480782

[B108] Okamoto T Matsuyama T Mori S Hamamoto Y Kobayashi N Yamamoto N Josephs SF Wong-Staal F Shimotohno K Augmentation of human immunodeficiency virus type 1 gene expression by tumor necrosis factor alpha AIDS Res Hum Retroviruses 1989 5 131 138 10.1089/aid.1989.5.131 2713164

[B109] Farrar WL Korner M Clouse KA Cytokine regulation of human immunodeficiency virus expression Cytokine 1991 3 531 542 10.1016/1043-4666(91)90479-W 1790301

[B110] Poli G Fauci AS The role of monocyte/macrophages and cytokines in the pathogenesis of HIV infection Pathobiology 1992 60 246 251 10.1159/000163729 1388722

[B111] Gregory CD Devitt A The macrophage and the apoptotic cell: an innate immune interaction viewed simplistically? Immunology 2004 113 1 14 10.1111/j.1365-2567.2004.01959.x 15312130 PMC1782541

[B112] Fadok VA Bratton DL Konowal A Freed PW Westcott JY Henson PM Macrophages that have ingested apoptotic cells in vitro inhibit proinflammatory cytokine production through autocrine/paracrine mechanisms involving TGF-beta, PGE2, and PAF J Clin Invest 1998 101 890 898 10.1172/JCI1112 9466984 PMC508637

[B113] Tassiulas I Park-Min KH Hu Y Kellerman L Mevorach D Ivashkiv LB Apoptotic cells inhibit LPS-induced cytokine and chemokine production and IFN responses in macrophages Hum Immunol 2007 68 156 164 10.1016/j.humimm.2006.12.008 17349870 PMC2736914

[B114] Chung EY Kim SJ Ma XJ Regulation of cytokine production during phagocytosis of apoptotic cells Cell Res 2006 16 154 161 10.1038/sj.cr.7310021 16474428

[B115] Alfano M Crotti A Vicenzi E Poli G New players in cytokine control of HIV infection Curr HIV/AIDS Rep 2008 5 27 32 10.1007/s11904-008-0005-5 18417032

[B116] Sozzani S Ghezzi S Iannolo G Luini W Borsatti A Polentarutti N Sica A Locati M Mackay C Wells TN Biswas P Vicenzi E Poli G Mantovani A Interleukin 10 increases CCR5 expression and HIV infection in human monocytes J Exp Med 1998 187 439 444 10.1084/jem.187.3.439 9449724 PMC2212126

[B117] Kutza J Crim L Feldman S Hayes MP Gruber M Beeler J Clouse KA Macrophage colony-stimulating factor antagonists inhibit replication of HIV-1 in human macrophages J Immunol 2000 164 4955 4960 10779806 10.4049/jimmunol.164.9.4955

[B118] Montaner LJ Griffin P Gordon S Interleukin-10 inhibits initial reverse transcription of human immunodeficiency virus type 1 and mediates a virostatic latent state in primary blood-derived human macrophages in vitro J Gen Virol 1994 75 3393 3400 10.1099/0022-1317-75-12-3393 7527834

[B119] Saville MW Taga K Foli A Broder S Tosato G Yarchoan R Interleukin-10 suppresses human immunodeficiency virus-1 replication in vitro in cells of the monocyte/macrophage lineage Blood 1994 83 3591 3599 7911340

[B120] Akridge RE Oyafuso LK Reed SG IL-10 is induced during HIV-1 infection and is capable of decreasing viral replication in human macrophages J Immunol 1994 153 5782 5789 7527449

[B121] Poli G Kinter AL Justement JS Bressler P Kehrl JH Fauci AS Transforming growth factor beta suppresses human immunodeficiency virus expression and replication in infected cells of the monocyte/macrophage lineage J Exp Med 1991 173 589 597 10.1084/jem.173.3.589 1705278 PMC2118806

[B122] Malim MH Natural resistance to HIV infection: The Vif-APOBEC interaction C R Biol 2006 329 871 875 10.1016/j.crvi.2006.01.012 17067930

[B123] Goila-Gaur R Strebel K HIV-1 Vif, APOBEC, and intrinsic immunity Retrovirology 2008 5 51 10.1186/1742-4690-5-51 18577210 PMC2443170

[B124] Franca R Spadari S Maga G APOBEC deaminases as cellular antiviral factors: a novel natural host defense mechanism Med Sci Monit 2006 12 RA92 98 16641889

[B125] Pillai SK Wong JK Barbour JD Turning up the volume on mutational pressure: is more of a good thing always better? (A case study of HIV-1 Vif and APOBEC3) Retrovirology 2008 5 26 10.1186/1742-4690-5-26 18339206 PMC2323022

[B126] Carthagena L Bergamaschi A Luna JM David A Uchil PD Margottin-Goguet F Mothes W Hazan U Transy C Pancino G Nisole S Human TRIM gene expression in response to interferons PLoS One 2009 4 e4894 10.1371/journal.pone.0004894 19290053 PMC2654144

[B127] Rajsbaum R Stoye JP O'Garra A Type I interferon-dependent and -independent expression of tripartite motif proteins in immune cells Eur J Immunol 2008 38 619 630 10.1002/eji.200737916 18286572

[B128] Towers GJ The control of viral infection by tripartite motif proteins and cyclophilin A Retrovirology 2007 4 40 10.1186/1742-4690-4-40 17565686 PMC1906832

[B129] Huthoff H Towers GJ Restriction of retroviral replication by APOBEC3G/F and TRIM5alpha Trends Microbiol 2008 16 612 619 10.1016/j.tim.2008.08.013 18976920 PMC3556578

[B130] Bishop KN Verma M Kim EY Wolinsky SM Malim MH APOBEC3G inhibits elongation of HIV-1 reverse transcripts PLoS Pathog 2008 4 e1000231 10.1371/journal.ppat.1000231 19057663 PMC2584787

[B131] Carthagena L Parise MC Ringeard M Chelbi-Alix MK Hazan U Nisole S Implication of TRIM alpha and TRIMCyp in interferon-induced anti-retroviral restriction activities Retrovirology 2008 5 59 10.1186/1742-4690-5-59 18613956 PMC2483995

[B132] Weissman D Poli G Fauci AS Interleukin 10 blocks HIV replication in macrophages by inhibiting the autocrine loop of tumor necrosis factor alpha and interleukin 6 induction of virus AIDS Res Hum Retroviruses 1994 10 1199 1206 10.1089/aid.1994.10.1199 7848677

[B133] Weissman D Poli G Fauci AS IL-10 synergizes with multiple cytokines in enhancing HIV production in cells of monocytic lineage J Acquir Immune Defic Syndr Hum Retrovirol 1995 9 442 449 7627621

[B134] Kootstra NA van 't Wout A Huisman HG Miedema F Schuitemaker H Interference of interleukin-10 with human immunodeficiency virus type 1 replication in primary monocyte-derived macrophages J Virol 1994 68 6967 6975 7933078 10.1128/jvi.68.11.6967-6975.1994 PMC237133

[B135] Yamamoto JK Barre-Sinoussi F Bolton V Pedersen NC Gardner MB Human alpha- and beta-interferon but not gamma-suppress the in vitro replication of LAV, HTLV-III, and ARV-2 J Interferon Res 1986 6 143 152 2425014 10.1089/jir.1986.6.143

[B136] Peng G Lei KJ Jin W Greenwell-Wild T Wahl SM Induction of APOBEC3 family proteins, a defensive maneuver underlying interferon-induced anti-HIV-1 activity J Exp Med 2006 203 41 46 10.1084/jem.20051512 16418394 PMC2118075

[B137] Mangino G Percario ZA Fiorucci G Vaccari G Manrique S Romeo G Federico M Geyer M Affabris E In vitro treatment of human monocytes/macrophages with myristoylated recombinant Nef of human immunodeficiency virus type 1 leads to the activation of mitogen-activated protein kinases, IkappaB kinases, and interferon regulatory factor 3 and to the release of beta interferon J Virol 2007 81 2777 2791 10.1128/JVI.01640-06 17182689 PMC1865981

[B138] Fakruddin JM Lempicki RA Gorelick RJ Yang J Adelsberger JW Garcia-Pineres AJ Pinto LA Lane HC Imamichi T Noninfectious papilloma virus-like particles inhibit HIV-1 replication: implications for immune control of HIV-1 infection by IL-27 Blood 2007 109 1841 1849 10.1182/blood-2006-02-001578 17068156 PMC1801045

[B139] Imamichi T Yang J Huang DW Brann TW Fullmer BA Adelsberger JW Lempicki RA Baseler MW Lane HC IL-27, a novel anti-HIV cytokine, activates multiple interferon-inducible genes in macrophages AIDS 2008 22 39 45 10.1097/QAD.0b013e3282f3356c 18090390

[B140] Greenwell-Wild T Vazquez N Jin W Rangel Z Munson PJ Wahl SM Interleukin-27 inhibition of HIV-1 involves an intermediate induction of type I interferon Blood 2009 114 1864 1874 10.1182/blood-2009-03-211540 19556424 PMC2738572

[B141] Harty RN Pitha PM Okumura A Antiviral Activity of Innate Immune Protein ISG15 J Innate Immun 2009 1 397 404 10.1159/000226245 19680460 PMC2725329

[B142] Okumura A Lu G Pitha-Rowe I Pitha PM Innate antiviral response targets HIV-1 release by the induction of ubiquitin-like protein ISG15 Proc Natl Acad Sci USA 2006 103 1440 1445 10.1073/pnas.0510518103 16434471 PMC1360585

[B143] Wahl SM Greenwell-Wild T Vazquez N HIV accomplices and adversaries in macrophage infection J Leukoc Biol 2006 80 973 983 10.1189/jlb.0306130 16908514

[B144] Espert L Degols G Lin YL Vincent T Benkirane M Mechti N Interferon-induced exonuclease ISG20 exhibits an antiviral activity against human immunodeficiency virus type 1 J Gen Virol 2005 86 2221 2229 10.1099/vir.0.81074-0 16033969

[B145] Barr SD Smiley JR Bushman FD The interferon response inhibits HIV particle production by induction of TRIM22 PLoS Pathog 2008 4 e1000007 10.1371/journal.ppat.1000007 18389079 PMC2279259

[B146] Lazdins JK Klimkait T Woods-Cook K Walker M Alteri E Cox D Cerletti N Shipman R Bilbe G McMaster G In vitro effect of transforming growth factor-beta on progression of HIV-1 infection in primary mononuclear phagocytes J Immunol 1991 147 1201 1207 1869819

[B147] Kamga I Kahi S Develioglu L Lichtner M Maranon C Deveau C Meyer L Goujard C Lebon P Sinet M Hosmalin A Type I interferon production is profoundly and transiently impaired in primary HIV-1 infection J Infect Dis 2005 192 303 310 10.1086/430931 15962225

[B148] Herbein G Khan KA Is HIV infection a TNF receptor signalling-driven disease? Trends Immunol 2008 29 61 67 10.1016/j.it.2007.10.008 18178131

[B149] Aukrust P Liabakk NB Muller F Lien E Espevik T Froland SS Serum levels of tumor necrosis factor-alpha (TNF alpha) and soluble TNF receptors in human immunodeficiency virus type 1 infection--correlations to clinical, immunologic, and virologic parameters J Infect Dis 1994 169 420 424 7906293 10.1093/infdis/169.2.420

[B150] Jacquot G Le Rouzic E David A Mazzolini J Bouchet J Bouaziz S Niedergang F Pancino G Benichou S Localization of HIV-1 Vpr to the nuclear envelope: impact on Vpr functions and virus replication in macrophages Retrovirology 2007 4 84 10.1186/1742-4690-4-84 18039376 PMC2211753

[B151] Kilareski EM Shah S Nonnemacher MR Wigdahl B Regulation of HIV-1 transcription in cells of the monocyte-macrophage lineage Retrovirology 2009 6 118 10.1186/1742-4690-6-118 20030845 PMC2805609

[B152] Herbeuval JP Nilsson J Boasso A Hardy AW Kruhlak MJ Anderson SA Dolan MJ Dy M Andersson J Shearer GM Differential expression of IFN-alpha and TRAIL/DR5 in lymphoid tissue of progressor versus nonprogressor HIV-1-infected patients Proc Natl Acad Sci USA 2006 103 7000 7005 10.1073/pnas.0600363103 16632604 PMC1444883

[B153] Meyers JH Justement JS Hallahan CW Blair ET Sun YA O'Shea MA Roby G Kottilil S Moir S Kovacs CM Chun TW Fauci AS Impact of HIV on cell survival and antiviral activity of plasmacytoid dendritic cells PLoS One 2007 2 e458 10.1371/journal.pone.0000458 17520017 PMC1866176

[B154] Nelms K Keegan AD Zamorano J Ryan JJ Paul WE The IL-4 receptor: signaling mechanisms and biologic functions Annu Rev Immunol 1999 17 701 738 10.1146/annurev.immunol.17.1.701 10358772

[B155] Papasavvas E Sun J Luo Q Moore EC Thiel B MacGregor RR Minty A Mounzer K Kostman JR Montaner LJ IL-13 acutely augments HIV-specific and recall responses from HIV-1-infected subjects in vitro by modulating monocytes J Immunol 2005 175 5532 5540 16210662 10.4049/jimmunol.175.8.5532

[B156] Benoit M Desnues B Mege JL Macrophage polarization in bacterial infections J Immunol 2008 181 3733 3739 18768823 10.4049/jimmunol.181.6.3733

[B157] Goldmann O von Kockritz-Blickwede M Holtje C Chhatwal GS Geffers R Medina E Transcriptome analysis of murine macrophages in response to infection with Streptococcus pyogenes reveals an unusual activation program Infect Immun 2007 75 4148 4157 10.1128/IAI.00181-07 17526748 PMC1951976

[B158] Mahlknecht U Herbein G Macrophages and T-cell apoptosis in HIV infection: a leading role for accessory cells? Trends Immunol 2001 22 256 260 10.1016/S1471-4906(01)01898-1 11323283

[B159] Varin A Mukhopadhyay S Herbein G Gordon S Alternative activation of macrophages by IL-4 impairs phagocytosis of pathogens but potentiates microbial-induced signalling and cytokine secretion Blood 2009 115 353 62 10.1182/blood-2009-08-236711 19880493 PMC2808158

[B160] Herbein G Mahlknecht U Batliwalla F Gregersen P Pappas T Butler J O'Brien WA Verdin E Apoptosis of CD8+ T cells is mediated by macrophages through interaction of HIV gp120 with chemokine receptor CXCR4 Nature 1998 395 189 194 10.1038/26026 9744279

[B161] Ogden CA Pound JD Batth BK Owens S Johannessen I Wood K Gregory CD Enhanced apoptotic cell clearance capacity and B cell survival factor production by IL-10-activated macrophages: implications for Burkitt's lymphoma J Immunol 2005 174 3015 3023 15728515 10.4049/jimmunol.174.5.3015

[B162] Byrne A Reen DJ Lipopolysaccharide induces rapid production of IL-10 by monocytes in the presence of apoptotic neutrophils J Immunol 2002 168 1968 1977 11823533 10.4049/jimmunol.168.4.1968

[B163] Clerici M Shearer GM A TH1-->TH2 switch is a critical step in the etiology of HIV infection Immunol Today 1993 14 107 111 10.1016/0167-5699(93)90208-3 8096699

[B164] Oleksyk TK Shrestha S Truelove AL Goedert JJ Donfield SM Phair J Mehta S O'Brien SJ Smith MW Extended IL10 haplotypes and their association with HIV progression to AIDS Genes Immun 2009 10 309 322 10.1038/gene.2009.9 19295541 PMC3664918

[B165] Vasilescu A Heath SC Ivanova R Hendel H Do H Mazoyer A Khadivpour E Goutalier FX Khalili K Rappaport J Lathrop GM Matsuda F Zagury JF Genomic analysis of Th1-Th2 cytokine genes in an AIDS cohort: identification of IL4 and IL10 haplotypes associated with the disease progression Genes Immun 2003 4 441 449 10.1038/sj.gene.6363983 12944981

[B166] Sandanger O Ryan L Bohnhorst J Iversen AC Husebye H Halaas O Landro L Aukrust P Froland SS Elson G Visintin A Øktedalen O Damås JK Sundan A Golenbock D Espevik T IL-10 enhances MD-2 and CD14 expression in monocytes and the proteins are increased and correlated in HIV-infected patients J Immunol 2009 182 588 595 19109192 10.4049/jimmunol.182.1.588

[B167] Creery D Angel JB Aucoin S Weiss W Cameron WD Diaz-Mitoma F Kumar A Nef protein of human immunodeficiency virus and lipopolysaccharide induce expression of CD14 on human monocytes through differential utilization of interleukin-10 Clin Diagn Lab Immunol 2002 9 1212 1221 12414752 10.1128/CDLI.9.6.1212-1221.2002 PMC130120

[B168] Andrade RM Lima PG Filho RG Hygino J Milczanowski SF Andrade AF Lauria C Brindeiro R Tanuri A Bento CA Interleukin-10-secreting CD4 cells from aged patients with AIDS decrease in-vitro HIV replication and tumour necrosis factor alpha production AIDS 2007 21 1763 1770 10.1097/QAD.0b013e3282ca83fa 17690575

[B169] Brooks DG Ha SJ Elsaesser H Sharpe AH Freeman GJ Oldstone MB IL-10 and PD-L1 operate through distinct pathways to suppress T-cell activity during persistent viral infection Proc Natl Acad Sci USA 2008 105 20428 20433 10.1073/pnas.0811139106 19075244 PMC2629263

[B170] Gupta S Boppana R Mishra GC Saha B Mitra D HIV-1 Tat suppresses gp120-specific T cell response in IL-10-dependent manner J Immunol 2008 180 79 88 18097007 10.4049/jimmunol.180.1.79

[B171] Fantuzzi L Belardelli F Gessani S Monocyte/macrophage-derived CC chemokines and their modulation by HIV-1 and cytokines: a complex network of interactions influencing viral replication and AIDS pathogenesis J Leukoc Biol 2003 74 719 725 10.1189/jlb.0403175 12960239

[B172] Rajasingh J Bord E Luedemann C Asai J Hamada H Thorne T Qin G Goukassian D Zhu Y Losordo DW Kishore R IL-10-induced TNF-alpha mRNA destabilization is mediated via IL-10 suppression of p38 MAP kinase activation and inhibition of HuR expression FASEB J 2006 20 2112 2114 10.1096/fj.06-6084fje 16935932

[B173] Wang Y Rice AP Interleukin-10 inhibits HIV-1 LTR-directed gene expression in human macrophages through the induction of cyclin T1 proteolysis Virology 2006 352 485 492 10.1016/j.virol.2006.05.013 16781761

[B174] Brenchley JM Schacker TW Ruff LE Price DA Taylor JH Beilman GJ Nguyen PL Khoruts A Larson M Haase AT Douek DC CD4+ T cell depletion during all stages of HIV disease occurs predominantly in the gastrointestinal tract J Exp Med 2004 200 749 759 10.1084/jem.20040874 15365096 PMC2211962

[B175] Guadalupe M Reay E Sankaran S Prindiville T Flamm J McNeil A Dandekar S Severe CD4+ T-cell depletion in gut lymphoid tissue during primary human immunodeficiency virus type 1 infection and substantial delay in restoration following highly active antiretroviral therapy J Virol 2003 77 11708 11717 10.1128/JVI.77.21.11708-11717.2003 14557656 PMC229357

[B176] Guadalupe M Sankaran S George MD Reay E Verhoeven D Shacklett BL Flamm J Wegelin J Prindiville T Dandekar S Viral suppression and immune restoration in the gastrointestinal mucosa of human immunodeficiency virus type 1-infected patients initiating therapy during primary or chronic infection J Virol 2006 80 8236 8247 10.1128/JVI.00120-06 16873279 PMC1563811

[B177] Li Q Duan L Estes JD Ma ZM Rourke T Wang Y Reilly C Carlis J Miller CJ Haase AT Peak SIV replication in resting memory CD4+ T cells depletes gut lamina propria CD4+ T cells Nature 2005 434 1148 1152 15793562 10.1038/nature03513

[B178] Mattapallil JJ Douek DC Hill B Nishimura Y Martin M Roederer M Massive infection and loss of memory CD4+ T cells in multiple tissues during acute SIV infection Nature 2005 434 1093 1097 10.1038/nature03501 15793563

[B179] Mehandru S Poles MA Tenner-Racz K Horowitz A Hurley A Hogan C Boden D Racz P Markowitz M Primary HIV-1 infection is associated with preferential depletion of CD4+ T lymphocytes from effector sites in the gastrointestinal tract J Exp Med 2004 200 761 770 10.1084/jem.20041196 15365095 PMC2211967

[B180] Mehandru S Poles MA Tenner-Racz K Manuelli V Jean-Pierre P Lopez P Shet A Low A Mohri H Boden D Racz P Markowitz M Mechanisms of gastrointestinal CD4+ T-cell depletion during acute and early human immunodeficiency virus type 1 infection J Virol 2007 81 599 612 10.1128/JVI.01739-06 17065209 PMC1797467

[B181] Smit-McBride Z Mattapallil JJ McChesney M Ferrick D Dandekar S Gastrointestinal T lymphocytes retain high potential for cytokine responses but have severe CD4(+) T-cell depletion at all stages of simian immunodeficiency virus infection compared to peripheral lymphocytes J Virol 1998 72 6646 6656 9658111 10.1128/jvi.72.8.6646-6656.1998 PMC109855

[B182] Veazey RS DeMaria M Chalifoux LV Shvetz DE Pauley DR Knight HL Rosenzweig M Johnson RP Desrosiers RC Lackner AA Gastrointestinal tract as a major site of CD4+ T cell depletion and viral replication in SIV infection Science 1998 280 427 431 10.1126/science.280.5362.427 9545219

[B183] Li L Meng G Graham MF Shaw GM Smith PD Intestinal macrophages display reduced permissiveness to human immunodeficiency virus 1 and decreased surface CCR5 Gastroenterology 1999 116 1043 1053 10.1016/S0016-5085(99)70007-7 10220496

[B184] Meng G Sellers MT Mosteller-Barnum M Rogers TS Shaw GM Smith PD Lamina propria lymphocytes, not macrophages, express CCR5 and CXCR4 and are the likely target cell for human immunodeficiency virus type 1 in the intestinal mucosa J Infect Dis 2000 182 785 791 10.1086/315790 10950772

[B185] Gorry PR Bristol G Zack JA Ritola K Swanstrom R Birch CJ Bell JE Bannert N Crawford K Wang H Schols D De Clercq E Kunstman K Wolinsky SM Gabuzda D Macrophage tropism of human immunodeficiency virus type 1 isolates from brain and lymphoid tissues predicts neurotropism independent of coreceptor specificity J Virol 2001 75 10073 10089 10.1128/JVI.75.21.10073-10089.2001 11581376 PMC114582

[B186] Smith PD Smythies LE Mosteller-Barnum M Sibley DA Russell MW Merger M Sellers MT Orenstein JM Shimada T Graham MF Kubagawa H Intestinal macrophages lack CD14 and CD89 and consequently are down-regulated for LPS- and IgA-mediated activities J Immunol 2001 167 2651 2656 11509607 10.4049/jimmunol.167.5.2651

[B187] Smythies LE Sellers M Clements RH Mosteller-Barnum M Meng G Benjamin WH Orenstein JM Smith PD Human intestinal macrophages display profound inflammatory anergy despite avid phagocytic and bacteriocidal activity J Clin Invest 2005 115 66 75 15630445 10.1172/JCI19229 PMC539188

[B188] Schenk M Bouchon A Birrer S Colonna M Mueller C Macrophages expressing triggering receptor expressed on myeloid cells-1 are underrepresented in the human intestine J Immunol 2005 174 517 524 15611278 10.4049/jimmunol.174.1.517

[B189] Schenk M Bouchon A Seibold F Mueller C TREM-1--expressing intestinal macrophages crucially amplify chronic inflammation in experimental colitis and inflammatory bowel diseases J Clin Invest 2007 117 3097 3106 10.1172/JCI30602 17853946 PMC1974863

[B190] Rugtveit J Bakka A Brandtzaeg P Differential distribution of B7.1 (CD80) and B7.2 (CD86) costimulatory molecules on mucosal macrophage subsets in human inflammatory bowel disease (IBD) Clin Exp Immunol 1997 110 104 113 10.1111/j.1365-2249.1997.507-ce1404.x 9353156 PMC1904794

[B191] Smith PD Meng G Salazar-Gonzalez JF Shaw GM Macrophage HIV-1 infection and the gastrointestinal tract reservoir J Leukoc Biol 2003 74 642 649 10.1189/jlb.0503219 12960227

[B192] Smith PD Ochsenbauer-Jambor C Smythies LE Intestinal macrophages: unique effector cells of the innate immune system Immunol Rev 2005 206 149 159 10.1111/j.0105-2896.2005.00288.x 16048547

[B193] Shen R Richter HE Clements RH Novak L Huff K Bimczok D Sankaran-Walters S Dandekar S Clapham PR Smythies LE Smith PD Macrophages in vaginal but not intestinal mucosa are monocyte-like and permissive to human immunodeficiency virus type 1 infection J Virol 2009 83 3258 3267 10.1128/JVI.01796-08 19153236 PMC2655566

[B194] Fischer-Smith T Bell C Croul S Lewis M Rappaport J Monocyte/macrophage trafficking in acquired immunodeficiency syndrome encephalitis: lessons from human and nonhuman primate studies J Neurovirol 2008 14 318 326 10.1080/13550280802132857 18780233 PMC2728912

[B195] Koenig S Gendelman HE Orenstein JM Dal Canto MC Pezeshkpour GH Yungbluth M Janotta F Aksamit A Martin MA Fauci AS Detection of AIDS virus in macrophages in brain tissue from AIDS patients with encephalopathy Science 1986 233 1089 1093 10.1126/science.3016903 3016903

[B196] Cherner M Masliah E Ellis RJ Marcotte TD Moore DJ Grant I Heaton RK Neurocognitive dysfunction predicts postmortem findings of HIV encephalitis Neurology 2002 59 1563 1567 12451198 10.1212/01.wnl.0000034175.11956.79

[B197] Nuovo GJ Alfieri ML AIDS dementia is associated with massive, activated HIV-1 infection and concomitant expression of several cytokines Mol Med 1996 2 358 366 10.1007/s0089460020358 8784788 PMC2230156

[B198] Saito Y Sharer LR Epstein LG Michaels J Mintz M Louder M Golding K Cvetkovich TA Blumberg BM Overexpression of nef as a marker for restricted HIV-1 infection of astrocytes in postmortem pediatric central nervous tissues Neurology 1994 44 474 481 8145918 10.1212/wnl.44.3_part_1.474

[B199] Ghafouri M Amini S Khalili K Sawaya BE HIV-1 associated dementia: symptoms and causes Retrovirology 2006 3 28 10.1186/1742-4690-3-28 16712719 PMC1513597

[B200] Davis LE Hjelle BL Miller VE Palmer DL Llewellyn AL Merlin TL Young SA Mills RG Wachsman W Wiley CA Early viral brain invasion in iatrogenic human immunodeficiency virus infection Neurology 1992 42 1736 1739 1513462 10.1212/wnl.42.9.1736

[B201] Niederkorn JY See no evil, hear no evil, do no evil: the lessons of immune privilege Nat Immunol 2006 7 354 359 10.1038/ni1328 16550198

[B202] Li MO Wan YY Sanjabi S Robertson AK Flavell RA Transforming growth factor-beta regulation of immune responses Annu Rev Immunol 2006 24 99 146 10.1146/annurev.immunol.24.021605.090737 16551245

[B203] Boche D Cunningham C Gauldie J Perry VH Transforming growth factor-beta 1-mediated neuroprotection against excitotoxic injury in vivo J Cereb Blood Flow Metab 2003 23 1174 1182 10.1097/01.WCB.0000090080.64176.44 14526228

[B204] Persidsky Y Poluektova L Immune privilege and HIV-1 persistence in the CNS Immunol Rev 2006 213 180 194 10.1111/j.1600-065X.2006.00440.x 16972904

[B205] Massa PT Specific suppression of major histocompatibility complex class I and class II genes in astrocytes by brain-enriched gangliosides J Exp Med 1993 178 1357 1363 10.1084/jem.178.4.1357 8376939 PMC2191216

[B206] Achim CL Morey MK Wiley CA Expression of major histocompatibility complex and HIV antigens within the brains of AIDS patients AIDS 1991 5 535 541 10.1097/00002030-199105000-00009 1863405

[B207] An SF Ciardi A Giometto B Scaravilli T Gray F Scaravilli F Investigation on the expression of major histocompatibility complex class II and cytokines and detection of HIV-1 DNA within brains of asymptomatic and symptomatic HIV-1-positive patients Acta Neuropathol 1996 91 494 503 10.1007/s004010050457 8740230

[B208] Persidsky Y Ghorpade A Rasmussen J Limoges J Liu XJ Stins M Fiala M Way D Kim KS Witte MH Weinand M Carhart L Gendelman HE Microglial and astrocyte chemokines regulate monocyte migration through the blood-brain barrier in human immunodeficiency virus-1 encephalitis Am J Pathol 1999 155 1599 1611 10550317 10.1016/S0002-9440(10)65476-4 PMC1866982

[B209] Suter T Biollaz G Gatto D Bernasconi L Herren T Reith W Fontana A The brain as an immune privileged site: dendritic cells of the central nervous system inhibit T cell activation Eur J Immunol 2003 33 2998 3006 10.1002/eji.200323611 14579268

[B210] Fassnacht U Ackermann A Staeheli P Hausmann J Immunization with dendritic cells can break immunological ignorance toward a persisting virus in the central nervous system and induce partial protection against intracerebral viral challenge J Gen Virol 2004 85 2379 2387 10.1099/vir.0.80115-0 15269380

[B211] Schwartz AJ Alvarez X Lackner AA Distribution and immunophenotype of DC-SIGN-expressing cells in SIV-infected and uninfected macaques AIDS Res Hum Retroviruses 2002 18 1021 1029 10.1089/08892220260235380 12396454

[B212] Gray F Lescs MC Keohane C Paraire F Marc B Durigon M Gherardi R Early brain changes in HIV infection: neuropathological study of 11 HIV seropositive, non-AIDS cases J Neuropathol Exp Neurol 1992 51 177 185 10.1097/00005072-199203000-00007 1538241

[B213] Potula R Poluektova L Knipe B Chrastil J Heilman D Dou H Takikawa O Munn DH Gendelman HE Persidsky Y Inhibition of indoleamine 2,3-dioxygenase (IDO) enhances elimination of virus-infected macrophages in an animal model of HIV-1 encephalitis Blood 2005 106 2382 2390 10.1182/blood-2005-04-1403 15961516 PMC1895260

[B214] McCrossan M Marsden M Carnie FW Minnis S Hansoti B Anthony IC Brettle RP Bell JE Simmonds P An immune control model for viral replication in the CNS during presymptomatic HIV infection 2006 129 503 516 16317019 10.1093/brain/awh695

[B215] Gray F Scaravilli F Everall I Chretien F An S Boche D Adle-Biassette H Wingertsmann L Durigon M Hurtrel B Chiodi F Bell J Lantos P Neuropathology of early HIV-1 infection Brain Pathol 1996 6 1 15 10.1111/j.1750-3639.1996.tb00775.x 8866743

[B216] Wingertsmann L Chretien F Authier FJ Paraire F Durigon M Gray F [Central nervous system lesions in the early stages of HIV infection] Arch Anat Cytol Pathol 1997 45 106 117 9382601

[B217] Sacktor N McDermott MP Marder K Schifitto G Selnes OA McArthur JC Stern Y Albert S Palumbo D Kieburtz K De Marcaida JA Cohen B Epstein L HIV-associated cognitive impairment before and after the advent of combination therapy J Neurovirol 2002 8 136 142 10.1080/13550280290049615 11935465

[B218] Wang T Gong N Liu J Kadiu I Kraft-Terry SD Mosley RL Volsky DJ Ciborowski P Gendelman HE Proteomic modeling for HIV-1 infected microglia-astrocyte crosstalk PLoS One 2008 3 e2507 10.1371/journal.pone.0002507 18575609 PMC2429966

[B219] Takahashi K Wesselingh SL Griffin DE McArthur JC Johnson RT Glass JD Localization of HIV-1 in human brain using polymerase chain reaction/in situ hybridization and immunocytochemistry Ann Neurol 1996 39 705 711 10.1002/ana.410390606 8651642

[B220] Wang Z Pekarskaya O Bencheikh M Chao W Gelbard HA Ghorpade A Rothstein JD Volsky DJ Reduced expression of glutamate transporter EAAT2 and impaired glutamate transport in human primary astrocytes exposed to HIV-1 or gp120 Virology 2003 312 60 73 10.1016/S0042-6822(03)00181-8 12890621

[B221] Fine SM Angel RA Perry SW Epstein LG Rothstein JD Dewhurst S Gelbard HA Tumor necrosis factor alpha inhibits glutamate uptake by primary human astrocytes. Implications for pathogenesis of HIV-1 dementia J Biol Chem 1996 271 15303 15306 10.1074/jbc.271.26.15303 8663435

[B222] Zhou BY Liu Y Kim B Xiao Y He JJ Astrocyte activation and dysfunction and neuron death by HIV-1 Tat expression in astrocytes Mol Cell Neurosci 2004 27 296 305 15519244 10.1016/j.mcn.2004.07.003

[B223] Garden GA Microglia in human immunodeficiency virus-associated neurodegeneration Glia 2002 40 240 251 10.1002/glia.10155 12379911

[B224] McArthur JC Brew BJ Nath A Neurological complications of HIV infection Lancet Neurol 2005 4 543 555 10.1016/S1474-4422(05)70165-4 16109361

[B225] Tyor WR Glass JD Griffin JW Becker PS McArthur JC Bezman L Griffin DE Cytokine expression in the brain during the acquired immunodeficiency syndrome Ann Neurol 1992 31 349 360 10.1002/ana.410310402 1586135

[B226] Wesselingh SL Takahashi K Glass JD McArthur JC Griffin JW Griffin DE Cellular localization of tumor necrosis factor mRNA in neurological tissue from HIV-infected patients by combined reverse transcriptase/polymerase chain reaction in situ hybridization and immunohistochemistry J Neuroimmunol 1997 74 1 8 10.1016/S0165-5728(96)00160-9 9119960

[B227] Xing HQ Moritoyo T Mori K Sugimoto C Ono F Izumo S Expression of proinflammatory cytokines and its relationship with virus infection in the brain of macaques inoculated with macrophage-tropic simian immunodeficiency virus Neuropathology 2009 29 13 19 10.1111/j.1440-1789.2008.00929.x 18507770

[B228] Cassol E Cassetta L Rizzi C Alfano M Poli G M1 and M2a polarization of human monocyte-derived macrophages inhibits HIV-1 replication by distinct mechanisms J Immunol 2009 182 6237 6246 10.4049/jimmunol.0803447 19414777

[B229] Boasso A Shearer GM Chougnet C Immune dysregulation in human immunodeficiency virus infection: know it, fix it, prevent it? J Intern Med 2009 265 78 96 10.1111/j.1365-2796.2008.02043.x 19093962 PMC2903738

